# Gender wage gap, quality of earnings and gender digital divide in the European context

**DOI:** 10.1007/s10663-022-09555-8

**Published:** 2022-12-01

**Authors:** Xose Picatoste, Anabela Mesquita, Fernando González-Laxe

**Affiliations:** 1 Department of Economics, Faculty of Economics and Business, University of A Coruña, A Coruña, Spain; 2 EDaSS Research Group on Economic Development and Social Sustainability, University Institute of Maritime Studies, Campus Elviña, s/n, 15071 A Coruña, Spain; 3 Instituto Superior de Contabilidade E Administração (ISCAP), Instituto Politécnico do Porto, Porto, Portugal; 4 University Institute of Maritime Studies, Campus Elviña, s/n, 15071 A Coruña, Spain

**Keywords:** Gender digital divide, Wage gap, Gender gap, Female employment, Work precariousness, Digital skills, J16, J78, J83, O30

## Abstract

One of the leading national and international objectives is to achieve more egalitarian societies. Avoiding gender or digital gaps are priorities generally assumed as concerns of governments and international organizations. This paper evaluates the digital gender divide in its three stages: access, use and results, relating it to gender and salary gaps in the context of the European Union. Cluster analysis was conducted to classify the countries according to their gender digital divide. The influence of age and studies level of males and females was revised. Based on the OECD and EUROSTAT data, an empirical analysis was conducted. By comparison of means, the significant variables influencing the gender digital divide were identified, probing that the educational level significantly influences it, especially in what refers to the third stage. Finally, through a regression analysis, it was proved that the influence of the insecurity and the gender wage gap goes beyond the women and affects society.

**Supplementary Information:**

The online version contains supplementary material available at 10.1007/s10663-022-09555-8.

## Introduction

The challenges posed by the fourth industrial revolution represent a new socioeconomic scenario that requires attention from institutions to continue along the path towards sustainable and inclusive growth prioritized in the 2030 Agenda (UN General Assembly 2015). Digitization can be an effective instrument to promote the inclusion of the most vulnerable social groups and facilitate gender equality. However, it is essential to anticipate possible adverse effects on women, particularly concerning stereotypes. To avoid a digital divide, it is essential to follow the guidelines marked by pillars II and III of the Digital Single Market Strategy. These pillars are related to creating a friendly environment that favours the development of digital networks and innovative services and guaranteeing the right of every citizen to participate in the benefits of an inclusive digital society. The European Parliament resolution of 28 April 2016 on gender equality and women's empowerment in the digital age (2015/2007(INI)) (European Parliament 2016) is a step forwards for women and all citizens to obtain the necessary skills to take advantage of internet opportunities and increase their chances of employment.

The changes related to the digitalized society have a particular impact on the labour market. Consequently, special attention must be paid to disadvantaged groups, such as women. For example, the current rate of women's participation in the world's labour force is close to 49%. In contrast, the rate for men is 75%. Thus, there is a difference of almost 26 percentage points, and in some regions, the disparity exceeds 50 percentage points (International Labour Office (ILO) 2022a). The new stage must boost the reduction of these differences.

Nevertheless, the risk of a gender digital divide (GDD) is associated with the present gender wage gap. In addition, women participate at a lower rate than men in the labour market and have more precarious jobs. The pandemic has increased inequality in various aspects, including gender disparities and the digital divide (which was accelerated by the pandemic), particularly for women and youth, for whom unemployment rates have increased. In terms of the global labour market impacts of the pandemic, young women have been among the worst affected. They have also been among the slowest groups to experience an improvement, aggravated by the barriers to re-entrance in the labour market (International Labour Office (ILO) 2022b).

This work seeks to clarify whether the digital gender gap affects success in the labour market in terms of income. This question is addressed from the perspective of the three stages affected by the digital divide (access, use and results), taking into account socioeconomic aspects such as the age and educational level of men and women.

The gender gap is assessed employing the Global Gender Gap Index (GGGI) based on the population-weighted average and calculated annually by the World Economic Forum. The index is calculated from four subindices, which collect information on economic participation and opportunities, educational attainment, health and survival, and political impoverishment. The score ranges from 0 to 1, and the closer to 1, the higher the equality. The GDD in 2021 was 67.7% considering 121 countries. However, this level increases to 68% when estimated only with the 107 countries that have been analysed continuously since 2006 (World Economic Forum 2021). The GDD is assessed in the framework of the European Declaration on Digital Rights and Principles for the Digital Decade (European Union 2022).

This paper aims to contribute to the analysis of GDD, focusing on the three stages of the digital divide (economic divide, usability divide, and empowerment divide, that is, access, use and outcomes). By a cluster and means comparison analysis and with data from OECD and EUROSTAT databases, empirical work was conducted to identify the main factors influencing such a divide in Europe and to determine the variables influencing the digital divide and the specific level affected by the digital divide. The structure of this paper is as follows: after the introduction section, a short summary of state of the art is given in section two; section three presents the methods, including the detailed objectives and hypotheses and some data; section four shows the results and discussion; and section five presents the conclusions and recommendations.

## The gender gap, digital divide and the labour market

Debates on the conceptualization of the digital divide have been developing and refining as information and communication technologies (ICT) have advanced. Most works have focused on the gaps in physical access to the use of ICT, the resources or access to the devices and the skills required for proper use. Other works have studied different groups (Arendt 2008), and the most recent studies described the three stages of the digital divide: access, use and outcomes (Liao et al. 2022; Wei et al. 2011).

The digital divide influences a wide range of economic, social and personal life spheres as well, from social to financial (Mohd Daud et al. 2021), labour market (Al Mamun and Wickremasinghe 2014) and relations with institutions, particularly government (Robinson et al. 2022). The issues targeted by digital divide studies are usually related to the internet. However, the terminology is diverse (Scheerder et al. 2017). In the case of the GDD, an additional nuance must be included: belonging to a specific social group with other simultaneous risks working together with gender. Thus, half of the population is in this situation by virtue of being women.

The GDD is sometimes linked to low educational levels (Hargittai 2010; Varela-Candamio et al. 2014) and rural areas (Caridad Sebastian and Ayuso García 2011; Novo-Corti et al. 2014; Novo-Corti and Baña Castro 2011; Whitacre and Mills 2010). This proves the importance of the technological development of the city of residence (Nieto-Mengotti et al. 2019). The academic literature has analysed the linkage of the GDD with access to the labour market (Picatoste et al. 2018a, b), especially for younger people. The GDD is linked to the stereotypes regarding the low interest of women in technical careers, taking into account their level of enrolment (Palomares-Ruiz et al. 2020). These attitudes are along the lines of the “technological deprivation” pointed out by Berrío Zapata et al. (2017). These practices are standard in different countries and usually continue into adolescence. Masanet, Gómez-Puertas and Pires (2021) analysed the GDD from a sociocultural perspective among adolescents in seven countries in Europe, Australia and South America. They found that this gap is related to gender stereotypes, for example, in media and video game practices (associated with boys) and the creation of stories (associated with girls) (Masanet et al. 2021). Thus, the gap is transversally present in different spaces, ages and levels of education, as evidenced in the research conducted in twelve Mexican public universities, with 3215 students, which shows that women have a more significant technological gap than men (Osorno Morales and Hernández Rivera 2021). Decreasing the gender gap is critical for social and labour inclusion (International Labour Office (ILO) 2008; UN Women 2021). Although disparities have decreased, they continue in employment (Losh 2004), and in addition, the GDD is a “phenomenon that constructs and legitimizes the exclusion of women from multiple knowledge activities, by naturalizing their “inability” to master the technological tools that today dominate the mediation of information flows and their effects” (Berrío Zapata et al. 2017).

### The gender divide

**Fig. 1:**
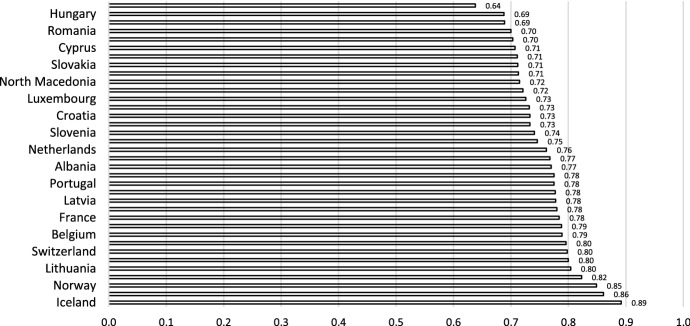
The global gender gap index. Source: Own elaboration from the World Economic Forum Global Gender Gap Report 2021 (World Economic Forum 2021)

In a generalized way, the gender gap is unacceptable in all its aspects, and national and international institutions have acknowledged this. Its evaluation has been approached from various perspectives. Figure 1 shows the data for one of the most widespread indices: the one offered by the World Economic Forum (World Economic Forum 2021).

The Global Gender Gap Index (World Economic Forum 2021) advances differently from country to country. However, with 2021 information, it is verified that these disparities may have been affected by the COVID-19 pandemic. The subindex evaluating political empowerment has stagnated, which is reflected in the value of the global indicator. The World Economic Forum assesses the estimated time to close the global gender gap at 135.6 years based on these results.

This indicator measures the extent to which countries have overcome the gender gap. It provides information about the gap that has been closed or the progress that has already been made towards parity.

The values range from 0 to 100, and the closer the values are to 100, the more fully the gender gap that has been closed (for example, 0.64 for Hungary and 0.89 for Iceland indicate the percentages of the gender gap that has been filled in these countries).

The calculation of this indicator has been carried out with the same methodology since 2006. Therefore, it is valuable for evaluating not only the gender gap but also the evolution of the progress made in closing it.

### The gender digital divide

The GDD must be interpreted in light of the transversal scope of gender equality assumed in the framework of Agenda 2030 (UN General Assembly 2015) and the European Declaration on Digital Rights and Principles for the Digital Decade (European Union 2022), in which the European Union declares the aim of improving people’s lives with the digital new era.

The three stages of the digital divide are found in all social groups, including women. Physical access to networks depends more on the characteristics of infrastructures and households than on individuals and, therefore, is free from gender aspects. However, in this work, the analysis of the three stages is relevant because the first stage (access) is related to the availability of the devices. People's access to these devices can show differences between men and women.

Reducing all digital divides is a priority, according to the European Union statements. In the first quarter of 2021, the European Commission presented a vision and pathways for the digital transformation of Europe by 2030, which was understood as a Digital Compass for the EU Digital Decade that evolves around four cardinal points: government, skills, infrastructures and business. Then, digital skills become crucial in this path (European Union 2021).

The summary of the Declaration on Digital Rights and Principles for the Digital Decade is shown in Table 1.

**Table 1:** Declaration on digital rights and principles for the digital decade. Source: Own elaboration from (European Union 2022)

Chapters	Commitments
Chapter I: putting people at the centre of the digital transformation	Strengthening the democratic framework for a digital transformation that benefits everyone and improves the lives of all Europeans Taking necessary measures to ensure that the values of the Union and the rights of individuals as recognized by Union law are respected online as well as offline Fostering responsible and diligent action by all digital actors, public and private, for a safe and secure digital environment Actively promoting this vision of the digital transformation, including in our international relations
Chapter II: solidarity and inclusion	Making sure that technological solutions respect people’s rights, enable their exercise and promote inclusion A digital transformation that leaves nobody behind. It should notably include elderly people, persons with disabilities, or marginalized, vulnerable or disenfranchised people and those who act on their behalf Developing adequate frameworks so that all market actors benefiting from the digital transformation assume their social responsibilities and make a fair and proportionate contribution to the costs of public goods, services and infrastructures, for the benefit of all Europeans Ensuring access to excellent connectivity for everyone, wherever they live and whatever their income Protecting a neutral and open internet where content, services, and applications are not unjustifiably blocked or degraded Promoting and supporting efforts to equip all education and training institutions with digital connectivity, infrastructure and tools Supporting efforts that allow learners and teachers to acquire and share all necessary digital skills and competences to take an active part in the economy, society, and in democratic processes Giving everyone the possibility to adjust to changes brought by the digitalization of work through upskilling and reskilling Ensuring that everyone shall be able to disconnect and benefit from safeguards for work–life balance in a digital environment Ensuring that all Europeans are offered an accessible, secure and trusted digital identity that gives access to a broad range of online services Ensuring wide accessibility and reuse of government information Facilitating and supporting seamless, secure and interoperable access across the Union to digital health and care services, including health records, designed to meet people’s needs
Chapter III: freedom of choice	Ensuring transparency about the use of algorithms and artificial intelligence and that people are empowered and informed when interacting with them Ensuring that algorithmic systems are based on suitable datasets to avoid unlawful discrimination and enable human supervision of outcomes affecting people Ensuring that technologies, such as algorithms and artificial intelligence, are not used to predetermine people’s choices, for example regarding health, education, employment, and their private life Providing for safeguards to ensure that artificial intelligence and digital systems are safe and used in full respect of people’s fundamental rights Ensuring a safe, secure and fair online environment where fundamental rights are protected and responsibilities of platforms, especially large players and gatekeepers, are well defined
Chapter IV: participation in the digital public space	Supporting the development and best use of digital technologies to stimulate citizen engagement and democratic participation Continuing safeguarding fundamental rights online, notably the freedom of expression and information Taking measures to tackle all forms of illegal content in proportion to the harm they can cause, and in full respect of the right to freedom of expression and information, and without establishing any general monitoring obligations Creating an online environment where people are protected against disinformation and other forms of harmful content
Chapter V: safety, security and empowerment	Protecting the interests of people, businesses and public institutions against cybercrime, including data breaches and cyberattacks. This includes protecting digital identity from identity theft or manipulation Countering and holding accountable those that seek to undermine security online and the integrity of the Europeans’ online environment or that promote violence and hatred through digital means Ensuring the possibility to easily move personal data between different digital services Promoting a positive, age-appropriate and safe digital environment for children and young people Providing opportunities to all children to acquire the necessary skills and competences to navigate the online environment actively and safely and make informed choices when online Protecting all children against harmful and illegal content, exploitation, manipulation and abuse online and preventing the digital space from being used to commit or facilitate crimes
Chapter VI: sustainability	Supporting the development and use of sustainable digital technologies that have minimal environmental and social impact Developing and deploying digital solutions with positive impact on the environment and climate

The EU points out its commitment to place people as the central axis of digital transformation, which must be promoted in public and private spheres (Chapter I), and to ensure respect for individual rights, protecting the most vulnerable groups and facilitating their inclusion. In this field, digital access is crucial, which is why universal connectivity is promoted, both in terms of access and skills (Chapter II). Chapter III emphasizes the importance of algorithms related to artificial intelligence to guarantee human rights and avoid discrimination or influence over people's options in health, education, employment, or their private life. Another critical issue for the EU is democratic participation (Chapter IV), which is intended to be strengthened through digital technologies in a secure environment that guarantees freedom of expression and protects against harmful content. The security and protection of the interests of people, companies and public institutions against cybercrimes and the protection of digital identity are addressed in Chapter V. These protections are contextualized for people according to their sociodemographic characteristics. Sustainability in general and environmental preservation, in particular, are constituted in a final chapter (VI) to underline that it is necessary to minimize the negative technological impacts on society and the environment.

### The precariousness of female employment

The precariousness of labour in general, and of women in particular, has been the subject of concern for policy-makers and academics, with exhaustive literature in various branches of knowledge and approaches. There is no unified definition of precarious work within and across the selected countries based on the information collected. Various definitions of precarious work are adopted in the literature, and they vary in terms of scope across countries. In particular, a comprehensive definition of precarious work is in place in France. The French National Institute for Statistics and Economic Studies (INSEE) defines as ‘precarious’ all types of work that are not covered by a permanent work contract, such as interim, fixed-term contracts, apprenticeships and state-aided contracts (Buckingham et al. 2021). Although the definition of precarious work is not well stated, a good reference is the one proposed by the European Institute for Gender Equality (EIGE): a job can be considered “precarious” when it shows at least one of these three conditions:

- Very low pay, where take-home pay from a worker’s main job is below the first quintile.
- Very low-intensity working hours (including mini-jobs and zero-hour contracts).
- Low job security (either a temporary contract or a permanent contract with a high risk of loss or termination).

The European Union is willing to advance in avoiding precariousness. This willingness was reflected in Directives (EU) 2019/1152 and (EU) 2019/1158 (European Parliament 2019a, b), the first of which dealt explicitly with precarious work for the first time and recommended its prevention while prohibiting the abuse of employment contracts that could induce it. The second of the directives refers to the quality of employment in terms of conciliation, which favours female labour inclusion. In addition to these rules, a network of actions has been woven to work in line with these rules. The aim is to eradicate job insecurity for all and, at the same time, promote equal employment for women, trying to eliminate prejudices and stereotypes, such as those that place women outside the sphere of studies or scientific occupations and technology, driving them away or discouraging them from participating in these labour spheres (Buckingham et al. 2021).

**Fig. 2:**
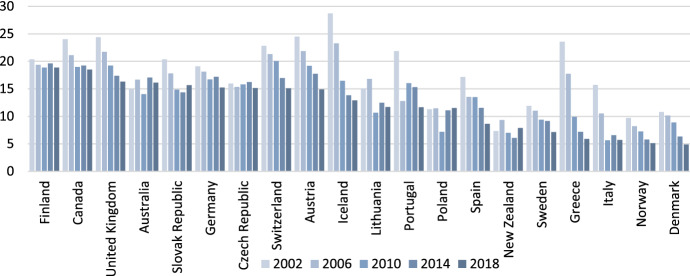
Gender wage gap evolution 2002–2018. Source: Own elaboration from OECD labour statistics

The differences between males and females in the labour market are extended to all spheres. The current rate of women's participation in the world's labour force is close to 49%, while the rate for men is 75%, a difference of 26 percentage points, and in some regions, the disparity exceeds 50 percentage points (International Labour Office (ILO) 2022b). In addition to participation, another important issue is the lower wages women receive for the same work. This so-called wage gap is being reduced but is still present worldwide. In Fig. 2, the values of this index are reflected for some countries (those with complete information for the series) for the years 2002, 2006, 2010, 2014 and 2018. These values are ordered from largest to smallest by the value of the gender wage gap in 2018. It can be seen how the gender wage gap, in general, diminishes over time. The lowest rates are for Demark and Norway. The gender wage gap, according to the OECD, is the difference between the median earnings of men and women relative to the median earnings of men. Data refer to full-time employees on the one hand and self-employed on the other (OECD 2022).

Some specific examples of the gender wage gap can give an idea of the situation in such countries and their evolution. For instance, the United States had a gender wage gap rate (for employed persons) in 2021 of 16.9%, compared to the gap in 2011 of 17.8% and that in 2001 of 23.6%. As seen, the gap is decreasing, but at a decreasing rate (in the decade from 2001 to 2011, it was reduced by 24.6% and in the decade from 2011 to 2021 by 5.5%). These data are hopeful for the path taken on the one hand, but on the other hand, they give an indicator of the time that remains to achieve equality. The trend is the same in the European Union (27); although starting from lower figures (16.8%, 12.5% and 10.3%), the reduction rates are similar: 25.6% and 17.6% for 2001 and 2021, respectively (OECD 2022).

**Fig. 3:**
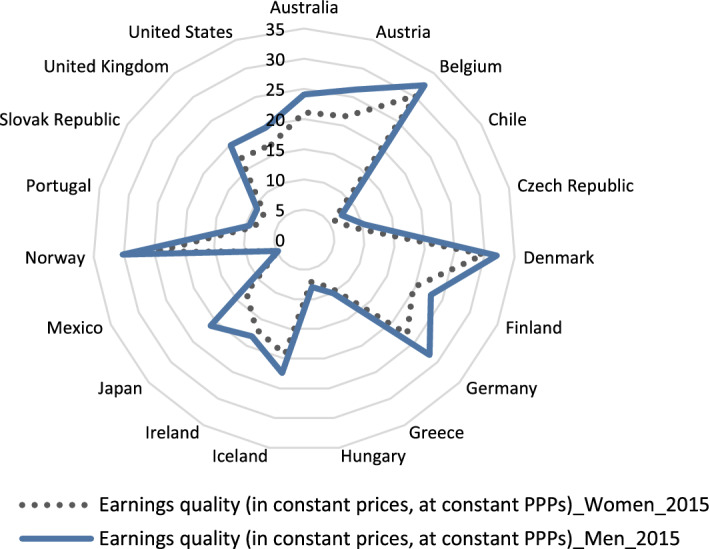
Earnings quality for men and women. Source: Own elaboration from OECD statistics: Job Quality Index

Regarding earnings quality, the OECD Job Quality Index provides a subindex assessing this item, which is calculated from the level and distribution of earnings, considering their importance for well-being; both indicators influence well-being, the first increasing satisfaction, and the other in the opposite way, due to efforts to reduce inequality in society, since people tend to display an inherent dislike of high inequality (OECD 2014). The results for this indicator for the most recently available data are shown for males and females in Fig. 3, where the dotted line, which represents women's earnings, is always inside the graph, which means that it takes lower values than the continuous line (representing men's earnings) with higher values. This graph clearly shows this generalized difference and, simultaneously, shows the countries with greater equality (where the lines are close, such as Norway and Belgium) and with greater differences (such as Greece and Mexico).

## Method and procedure

The model developed to explain the earnings quality is based on the relationship of this quality with the gender gap and the security of maintaining employment (reflected by the conditions of the labour market), as expressed in Eq. 1, where ${y}_{t}$ represents the quality of the earnings and ${x}_{1}$ and ${x}_{2}$ the insecurity in the labour market and the gender wage gap, respectively. It is expected that the higher the insecurity is, the lower the earnings quality, and the higher the gender wage gap is, the lower the earnings quality. Data analysis will test these hypotheses to determine their acceptance or rejection.

1 $$y={\beta }_{0}+{\beta }_{1}{x}_{1}+{\beta }_{2}{x}_{2}+\varepsilon$$

Considering the different possible sources of inequalities that can occur for women, the objectives and hypotheses of this work can be systematized as follows (Table 2):

**Table 2:** Objectives and hypotheses

Objective	Hypothesis
Determining the main significant socioeconomic characteristics to explain the GDD in Europe	H1a: age is a relevant variable in explaining the GDD H1b: educational level is a relevant variable in explaining the GDD
Analysing the digital divide at the three levels depending on the educational level	H2a: there is a GDD at level 1 H2b: there is a GDD at level 2 H2c: there is a GDD at level 3
Does the GDD affect success in the labour market in terms of earnings?	H3a: insecurity in the labour market negatively influences the quality of earnings for women H3b: the gender wage gap negatively influences the quality of earnings for women

Objective 1: Determining the main significant socioeconomic characteristics to explain the GDD in Europe.

### H1a

Age is a relevant variable in explaining GDD.

### H1b

Educational level is a relevant variable in explaining GDD.

Objective 2: Analysing the digital divide at the three levels depending on the educational level.

### H2a

There is a GDD at level 1.

### H2b

There is a GDD at level 2.

### H2c

There is a GDD at level 3.

Objective 3: Does the GDD affect success in the labour market in terms of earnings?

### H3a

Insecurity in the labour market negatively influences the quality of earnings for women.

### H3b

The gender wage gap negatively influences the quality of earnings for women.

Multiple factors affect the GDD. The World Health Organization states that “gender” refers to the socially constructed roles, behaviours, activities and attributes that a given society considers appropriate for men and women, thus creating the categories of “masculine” and “feminine” gender (World Health Organization 2015). Different gaps or divides linked to these concepts may arise, as is the case of the GDD. Gender equality is a right. The current situation requires urgent action to achieve economic, environmental and social sustainability, understood as a whole (Brundtland 1987). Amid a technological revolution, with disruptive changes, gender discrimination becomes an additional obstacle (UN Women 2021). Additionally, Agenda 2030 (UN General Assembly 2015) raises the need to achieve gender equality and empower all women and girls (Sustainable Development Goal 5). In general, a gender problem is detected in the shortage of women ICT specialists, but women cannot be excluded from access to technology because it is their right and it increases their digital skills, which are positively related to a higher probability of working with a permanent contract (Martínez Cantos et al. 2020). In this process, institutions are crucial (OXFAM Intermón 2019). The persistence of gender inequality was proven, for example, in a study of thirty-nine countries (Drabowicz 2014). Due to the impact of the pandemic, the digital divide has widened. The ILO estimates that although the effects of the pandemic on female employment will diminish in the future, a considerable gap is expected to remain for the time being. The most significant inequalities are found in upper-middle-income countries, where the employment rate for women in 2022 is expected to be 1.8 percentage points lower than in 2019, compared to a difference of only 1.6 percentage points for men, even though women have an employment rate 16 percentage points below that of men (International Labour Office (ILO) 2022b).

Together with Directives (EU) 2019/1152 and (EU) 2019/1158 (European Parliament 2019a, b) to avoid those inequalities, the European Union is undertaking other actions. For example, it launched the European skills agenda for sustainable competitiveness, social fairness and resilience, or the Digital Europe Programme, to increase the proportion of women graduating in STEM subjects and reduce the underrepresention of women in ICT-related sectors (European Union 2020b). Another initiative is the European Gender Equality Strategy for 2020–2025, which was established to eradicate gender stereotypes and close gender gaps, particularly in the labour market, specifically salary and pension gaps. It also set an objective of achieving the equitable participation of women in different sectors of the economy (European Union 2020a).

An empirical analysis was conducted to address the objectives set out in this paper and test the hypotheses. Data were collected from both the OECD and EUROSTAT. Some descriptive statistical procedures were applied, together with a complimentary analysis concerning digitization of the countries, through a hierarchical cluster classification analysis. To detect and evaluate possible differences between men and women, an analysis of a comparison of means for independent samples was carried out for all three areas of digitization. In addition, Levene's tests were calculated to check the homogeneity of variances in the groups under study beforehand. Finally, linear OLS regression analysis was used to test for the existence of causal relationships between digital skills and the labour market. These statistical and econometric analyses were carried out using the IBM SPSS statistical package, version 26. The secondary data collected from OECD and EUROSTAT were the source information for the econometric Eq. (1) and for testing the hypotheses. The sources are described in each specific analysis.

The three stages of the digital divide were estimated by the indicators shown in Table 3.

**Table 3:** Indicators for the three stages of the digital divide. Source: own elaboration. Data source: EUROSTAT

Stage 1—Access	Stage 2—Use (capability)	Stage 3—Outcome (competences?)
Access to the internet using a desktop		
Access to the internet using a laptop	Access to the internet in the last three months	Submitting completed forms (last 12 months)
Access to the internet using a tablet		Individuals with above basic overall digital skills (all five component indicators are at above basic level)
Access to the internet using a phone	Access to the internet daily (among the users of the internet in the last three months)	Employed with ICT education
Access to the internet using a mobile device		

To differentiate by gender, data on individuals are needed, as household data are not helpful. Therefore, to assess access to technology (the first stage of the digital divide), information on people who have never accessed the internet is used. Additionally, considered among the valuable indicators for this first stage are those referring to the type of device used to access the internet (fixed computer, laptop, tablet, smartphone or mobile devices) (see Table 3). For the second stage, use levels were considered in terms of frequent and daily access. The indicators used were extracted from EUROSTAT data in the section Science, technology, digital society, digital economy and society ICT usage in households and by individual internet users and the users of the internet daily [ISOC_CI_IFP_FU] (see Table 3).

For the assessment of the third stage, two indicators were used (see Table 3). The first indicator refers to the percentage of individuals who have sent forms to the institutions, duly filled in and completed, through the internet, in the last 12 months, whose data can be found in EUROSTAT in the section Science, technology, digital society, digital economy and society ICT usage in households and by individuals in E-government, namely, E-government activities of individuals via websites [ISOC_CIEGI_AC]. The second indicator is related to high digital skills, as indicated by the percentage of individuals with levels above basic overall digital skills, in all indicators, i.e., in the areas of 1. Information and data literacy skills, 2. Communication and collaboration skills, 3. Digital content creation skills, 4. Safety skills and 5. Problem-solving skills. The corresponding data have been collected from EUROSTAT in the section Science, technology, digital society, digital economy and society, digital skills, ICT users, individuals' level of digital skills (from 2021 onwards) [ISOC_SK_DSKL_I21] and individuals' level of digital skills (until 2019) [ISOC_SK_DSKL_I]. Additionally, the individuals employed with ICT education were included, with data from EUROSTAT Science, technology, digital society, digital economy and society, digital skills, ICT training, employed persons with ICT education by gender [ISOC_SKI_ITSEX].

The third objective will be assessed by employing OLS regression from data extracted from the OECD statistics database, where the gender wage gap at the median was the value assumed as the measurement of the gender wage gap, and the quality of earnings and insecurity at work were taken from the subindex of the Job Quality Index of the OECD. The relationship among the three variables explained in Eq. (1) will be tested.

## The digital skills, use and outcome of women and men in Europe

Prior to the analysis of the variables influencing the digital divide from this three-stage perspective, an overview of the differences among countries was performed. The data extracted from EUROSTAT focused on women’s and men’s access to the internet.

**Fig. 4:**
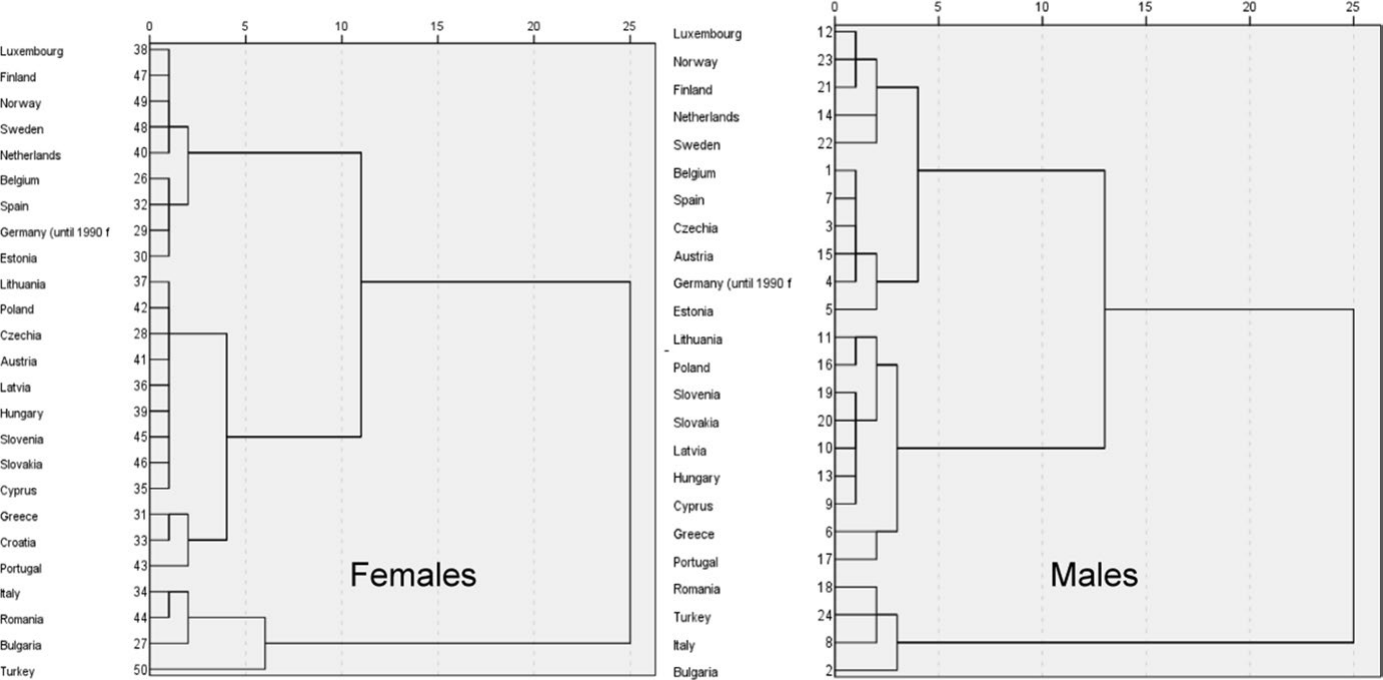
Cluster analysis for the digital divide (males and males and females)

Before analysing the variables affecting the digital divide from the perspective of its three stages, an overview of the differences between countries is presented, taking into account gender differences. The grouping of the countries was performed through hierarchical clustering according to Ward's method using the squared Euclidean distance and standardized values. The results obtained for the data for men and women, shown in Fig. 4, allow us to verify two aspects: On the one hand, the grouping of the countries, in general terms (if two groups are considered), is similar in both cases, but this grouping changes if we refine the adjustment and use sets of three or more groups, indicating gender differences. Specifically, if two groups are considered, there is a larger and a smaller group, which includes Bulgaria, Italy, Romania and Turkey, whose gender gap indices in 2021, according to the WEF report, are 0.746, 0.721, 0.700 and 0.638, respectively; from the more equalitarian to the less equalitarian, these countries are ranked 38th, 63rd, 88th and 133rd, respectively. The reference values are as follows: the minimum value reported is 0.444 in Afghanistan (ranked 156th, the most significant gender gap reported), and the maximum is 0.892 in Iceland (ranked 1st, the lowest gender gap reported) (World Economic Forum 2021).

The first objective of this work and the contrasting of the first and second hypotheses were accomplished with a sample of people who had never used the internet from 2016 to 2021. This sample was segmented by age and educational level and distinguished between men and women. The source was EUROSTAT Science (Science, technology, digital society, digital economy and society ICT usage in households and by individuals, connection to the internet and computer use; individuals—devices used to access the internet [ISOC_BDEK_DI]). Levene’s test and mean comparison analysis for independent samples were performed to determine whether there were significant differences between men and women. The results indicate that there are no differences in the mean age ratio that are related to age or educational level when the first stage is analysed, that is, access to technology. At this point, the detailed results are shown in the Online Appendix section, specifically in Tables A-1: Testing H/Stage 1: never access the internet, variable: Age (regarding age), and Stage 1: never access to the internet, variable: Education (regarding education). For the first analysis (age), 56 items were studied according to different age intervals and different years, and no significant differences were found for any of them; therefore, age cannot be considered as a differential reason. For the education analysis, the same result of no significant differences was found in all of the items analysed.

When the second stage was analysed, no differences were found for age reasons, but significant differences (*p* value < 0.001 for Student’s *t* test) were found regarding high educational levels. The specific results are shown in the Online Appendix section, specifically in Tables A-1: Testing H1 “Stages 2 and 3, access once a week to the internet, variable: age” for age and “Stages 2 and 3, access once a week to the internet, variable: education” for education level. For the age variable, no significant differences were found for any of the 56 items analysed. For educational level, there were significant differences (*p* value < 0.001) for all seven items related to “High Formal Education” for all analysed years (from 2015 to 2021) (in red in the Online Appendix). Thus, whilst age differences are not stated as relevant variables for the gender digital divide, educational level seems to be significant. Hypothesis H1a (age is a relevant variable in explaining the GDD) should be rejected, and Hypothesis H1b (educational level is a relevant variable in explaining the GDD) must be accepted.

The importance of the educational level in the digital divide was considered in the subsequent analysis. The database was reconstructed by taking the EUROSTAT variables explained in Table 3. It was broken down by academic level and gender for 2016, 2018, and 2021. Moreover, only the countries belonging to the European Union were considered in the analysis (to obtain all the detailed information needed). A comparison of means was performed for the three stages of the digital divide, and the results show that no statistically significant differences are found in the first and second stages. Specific results are provided in the Online Appendix in Tables A-2: Testing H2 “H2 (a) Stage 1, H2 (b) Stage 2 and H2 (c) Stage 3”. For Stage 2, no significant differences were found, and for Stage 1, significant differences (highlighted in red) were found only for people using a desktop, but no significant differences were found for the use of laptops, tablets or smartphones to access the internet; therefore, this factor must not be related to the access itself but to the specific device. At the same time, there are significant differences in the third stage for higher education and higher digital skills. The hypothesis test results lead to the rejection of H2a (there is a GDD at level 1) and H2b (there is a GDD at level 2) and the acceptance of hypothesis H2c (there is a GDD at level 3).

As seen in Table 4, the results obtained when estimating Eq. (1) with the OECD data are consistent with the a priori assumptions, both when using the pooled sample (Eq. 2) and when considering subsamples of males and females (Eqs. 3 and 4).

**Table 4:** Results of the regression analysis

Group	${b}_{0}$	${b}_{1}$	${b}_{2}$
All individuals^(a)^	37.826	− 2.730	− 0.580
	(*p* < 0.001)	(− 0.649)	(− 0.513)
		(*p* = 0.001)	(*p* = 0.007)
Male^(b)^	43.229	− 3.733	− 0.550
	(*p* < 0.001)	(− 0.638)	(− 0.459)
		(*p* = 0.005)	(*p* = 0.031)
Female^(c)^	33.830	− 1.851	− 0.620
	(*p* < 0.001)	(− 0.645)	(− 0.567)
		(*p* = 0.002)	(*p* = 0.004)

^(a)^R^2^ = 0.569, ^(b)^R^2^ = 0.505, ^(c)^R^2^ = 0.628

Estimation Method: OLS

The results in the brackets show the value of the standardized coefficients and the *p* values

According to Table 4, more than 50% of the dependent variable ${y}_{t}$ (quality of earnings) is explained by ${x}_{1}$ (insecurity in the labour market) and ${x}_{2}$ (gender wage gap), and both exogenous variables negatively influence the quality of earnings for males and females. Although the wage gender gap has a slightly greater influence for females (a coefficient of 0.620 for females and 0.550 for males), it is estimated that the influences of both insecurity in the labour market and the gender wage gap are significant and have a negative effect for both subsamples and thus for the total population. This indicates not only that women are affected but also that both variables must be taken into account in terms of the welfare of society as a whole. Ceteris paribus, if insecurity is reduced, men's earnings quality is estimated to increase more, while if the gender pay gap is reduced, women's earnings quality is estimated to increase more, but both variables are shown to be relevant in the estimations with the two subgroups of observations.

2 $${\widehat{y}}_{total}=37.826-2.730 {x}_{1}-0.580 {x}_{2}$$

3 $${\widehat{y}}_{male}=43.229-3.733{ x}_{1}-0.550 {x}_{2}$$

4 $${\widehat{y}}_{female}=33.830-1.851{ x}_{1}-0.620 {x}_{2}$$

The third objective of this work was achieved by testing H3a (the insecurity in the labour market negatively influences the quality of earnings for women) and H3b (the gender wage gap negatively influences the quality of earnings for women), according to the regression analysis results. A summary of the results of the test hypotheses is shown in Table 5 and Eq. (4).

**Table 5:** Results of hypothesis testing

Hypothesis	Result of hypothesis testing for the data analysed
H1a: age is a relevant variable in explaining the GDD	Rejected
H1b: educational level is a relevant variable in explaining the GDD	Accepted
H2a: there is a GDD at level 1	Rejected
H2b: there is a GDD at level 2	Rejected
H2c: there is a GDD at level 3	Accepted
H3a: the insecurity in the labour market negatively influences the quality of earnings for women	Accepted
H3b: the gender wage gap negatively influences the quality of earnings for women	Accepted

The results go beyond our expected findings since the tested hypotheses referred only to females, but our research results indicate that the effect is also observed for males. These results are consistent with the evidence that inequality affects not only women’s status (International Labour Office (ILO), 2008; UN Women, 2021) but also society as a whole.

In this work, variables have been included according to a transversal approach, in which education, age and access to technology (in its three phases) have been considered, together with the gender wage gap, security in employment and the digital divide. We have found academic works that analyse these aspects, but to the best of our knowledge, none have provided a multidimensional vision like the one in this work. For example, we have seen research on earnings and gender differences (Atkinson et al. 2018), job insecurity, digital skills (Losh 2004), and the digital gender gap (Ma 2022); however, we have not found works that seek explanations from a multidimensional aspect, such as the one proposed here.

The limitations of this research are mainly related to the data availability since it is an empirical contribution. Nevertheless, more countries and regions can be explored to confirm and generalise our results. Therefore, other lines of research on this topic are open: on the one hand, to extend the analysis to other geographic areas and, on the other hand, to continue deepening the influence of the analysed gaps in both genders. Since the results point to the impact of the gender wage gap on the quality of earnings of all society and, particularly, on males’ earnings, analysing the effects of the gender wage gap in males is of interest.

## Conclusions

The gender gap exists in all countries for which data are available. Although this gap is narrowing (according to the WEF indicator), it is necessary to continue promoting policies to reduce these differences.

The gender digital divide is generally decreasing, although progress is slower than desired. Moreover, the emergence of technologies targeted at robotization and artificial intelligence must be carefully considered due to its possible implications, especially in the most vulnerable groups.

Educational levels are relevant when analysing the digital gender gap. In addition, age does not seem relevant in the first two stages of the digital divide. However, it needs to be underlined that the first-stage data for physical access are general for households. Nevertheless, access to the devices depends on individuals, and it was analysed from a gender perspective.

The third stage of the digital divide, related to the outcomes, is the one that influences the digital gender divide. Although the underlying reasons that can explain this result are beyond the scope of this paper, it seems that they are based more on qualitative factors linked to stereotypes than on accurate data of the type used here.

Regarding the influence of the gender wage gap on the quality of earnings, although women suffer the most, it also affects men and, therefore, the whole of society. The results of this work mark the importance of the dynamics of incorporating technological progress for achieving gender equality and reducing economic inequalities and other gaps that damage social welfare.

This paper provides empirical evidence that the gender gap affects society as a whole and, in particular, men, beyond the fact that women feel it the most. The main novelty of this work is its consideration of the quality of the earnings received and how this factor relates to the gender gap in earnings and job security, issues suggested by previous research but not related jointly. Conceptually, the focus is on qualitative aspects related to decent work and the need for sustainable employment, along the lines proposed by the SDGs (Goal 8), reducing inequalities (Goal 10) and gender equality (Goal 5) within the general context of the framework of the 2030 Agenda. Methodologically, various analyses have been used, ranging from clustering to the regression by ordinary least squares, through an analysis of comparison of means, which has allowed the problem to be analysed from different points of view. In addition, two pillars have been combined: access to technology in its three phases, on the one hand, and the sociodemographic characteristics of age and education, in addition to gender. In this way, the study of a multidimensional problem is approached broadly. Based on these characteristics, the results of this research work can be helpful in the approach and design of social and labour policies to achieve a more inclusive and sustainable society, both from a social and economic point of view.

The results and the above reflections point to coherent and coordinated responses from public policies, which must be urgently demanded. These initiatives must encompass inclusive and equalitarian social and economic measures to protect employment and guarantee the continuity of productive activity and the income that allows individuals and households to face the challenges encountered during the current crisis, especially the most vulnerable groups and sectors and people most at risk of losing jobs and other means of livelihood. By taking these actions, the states will comply with their commitments regarding the 2030 Agenda and with the guidelines of the European Union while following the path of equality and the search for a more inclusive society to improve well-being. Applying these measures in the context of a permanent technological revolution, especially in the face of the challenges of artificial intelligence, becomes a central axis to achieve sustainable development without leaving anyone behind, as the European Union intends.

## Supplementary Information

Below is the link to the electronic supplementary material.

Supplementary file1 (DOCX 168 kb)
