# Supplementary material for: Gender wage gap, quality of earnings and gender digital divide in the European context
Source: Empirica (Dordr). 2022 Dec 1;50(2):301–21. doi: 10.1007/s10663-022-09555-8 (PMC9714396; doi:10.1007/s10663-022-09555-8)
Supplement: Supplementary file 1 — Supplementary file1 (DOCX 168 kb) [file 10663_2022_9555_MOESM1_ESM.docx]

# Appendix

## Tables A-1: Testing H1

### Stage 1: never access the Internet / Variable: Age

| **Test for independent samples** | | | | | | | | | | |
| --- | --- | --- | --- | --- | --- | --- | --- | --- | --- | --- |
|  | | Levene’s test of equality of variances | | t test for mean equality | | | | | | |
|  |  | F | Sig. | t | df | Sig. (two-tailed) | Means difference | Difference standard error | 95% confidence interval of the difference | |
|  |  |  |  |  |  |  |  |  | Lower | Upper |
| Age_16_19_2015 | Assuming equal variances | 1,195 | ,278 | -,451 | 66 | ,653 | -,41500 | ,91943 | -2,25069 | 1,42070 |
|  | No Assuming equal variances |  |  | -,469 | 61,714 | ,641 | -,41500 | ,88483 | -2,18391 | 1,35392 |
| Age_16_19_2016 | Assuming equal variances | 1,399 | ,241 | -,189 | 65 | ,851 | -,16129 | ,85431 | -1,86746 | 1,54488 |
|  | No Assuming equal variances |  |  | -,195 | 59,054 | ,846 | -,16129 | ,82539 | -1,81285 | 1,49027 |
| Age_16_19_2017 | Assuming equal variances | 3,226 | ,077 | -,944 | 71 | ,348 | -,63964 | ,67769 | -1,99091 | ,71163 |
|  | No Assuming equal variances |  |  | -,949 | 62,692 | ,346 | -,63964 | ,67403 | -1,98671 | ,70743 |
| Age_16_19_2018 | Assuming equal variances | 5,301 | ,024 | -1,345 | 73 | ,183 | -,84353 | ,62712 | -2,09338 | ,40632 |
|  | No Assuming equal variances |  |  | -1,355 | 56,654 | ,181 | -,84353 | ,62239 | -2,09001 | ,40296 |
| Age_16_19_2019 | Assuming equal variances | 2,690 | ,105 | -,940 | 71 | ,350 | -,49249 | ,52386 | -1,53703 | ,55205 |
|  | No Assuming equal variances |  |  | -,947 | 57,173 | ,348 | -,49249 | ,52012 | -1,53394 | ,54895 |
| Age_16_19_2020 | Assuming equal variances | ,486 | ,488 | -,177 | 69 | ,860 | -,08175 | ,46096 | -1,00134 | ,83785 |
|  | No Assuming equal variances |  |  | -,176 | 58,596 | ,861 | -,08175 | ,46357 | -1,00949 | ,84599 |
| Age_16_19_2021 | Assuming equal variances | 1,801 | ,184 | -,205 | 68 | ,838 | -,05714 | ,27917 | -,61422 | ,49993 |
|  | No Assuming equal variances |  |  | -,205 | 63,865 | ,838 | -,05714 | ,27917 | -,61487 | ,50058 |
| Age_16_24_2015 | Assuming equal variances | ,608 | ,438 | -,309 | 62 | ,758 | -,31250 | 1,01112 | -2,33370 | 1,70870 |
|  | No Assuming equal variances |  |  | -,309 | 51,202 | ,759 | -,31250 | 1,01112 | -2,34222 | 1,71722 |
| Age_16_24_2016 | Assuming equal variances | ,227 | ,636 | -,279 | 60 | ,781 | -,22581 | ,80950 | -1,84505 | 1,39343 |
|  | No Assuming equal variances |  |  | -,279 | 51,323 | ,781 | -,22581 | ,80950 | -1,85070 | 1,39908 |
| Age_16_24_2017 | Assuming equal variances | ,661 | ,419 | -,580 | 70 | ,564 | -,33333 | ,57502 | -1,48017 | ,81350 |
|  | No Assuming equal variances |  |  | -,580 | 66,377 | ,564 | -,33333 | ,57502 | -1,48127 | ,81460 |
| Age_16_24_2018 | Assuming equal variances | 4,168 | ,045 | -1,024 | 74 | ,309 | -,50000 | ,48826 | -1,47287 | ,47287 |
|  | No Assuming equal variances |  |  | -1,024 | 58,220 | ,310 | -,50000 | ,48826 | -1,47728 | ,47728 |
| Age_16_24_2019 | Assuming equal variances | 1,897 | ,173 | -,461 | 74 | ,646 | -,18421 | ,39936 | -,97995 | ,61152 |
|  | No Assuming equal variances |  |  | -,461 | 60,993 | ,646 | -,18421 | ,39936 | -,98278 | ,61435 |
| Age_16_24_2020 | Assuming equal variances | 2,164 | ,146 | -,528 | 70 | ,599 | -,19444 | ,36801 | -,92841 | ,53952 |
|  | No Assuming equal variances |  |  | -,528 | 58,122 | ,599 | -,19444 | ,36801 | -,93105 | ,54216 |
| Age_16_24_2021 | Assuming equal variances | ,979 | ,326 | ,620 | 68 | ,537 | ,17143 | ,27632 | -,37995 | ,72281 |
|  | No Assuming equal variances |  |  | ,620 | 66,653 | ,537 | ,17143 | ,27632 | -,38015 | ,72301 |
| Age_16_29_2015 | Assuming equal variances | ,856 | ,358 | -,521 | 62 | ,604 | -,56250 | 1,07982 | -2,72103 | 1,59603 |
|  | No Assuming equal variances |  |  | -,521 | 50,508 | ,605 | -,56250 | 1,07982 | -2,73085 | 1,60585 |
| Age_16_29_2016 | Assuming equal variances | ,927 | ,340 | -,446 | 60 | ,657 | -,38710 | ,86718 | -2,12171 | 1,34751 |
|  | No Assuming equal variances |  |  | -,446 | 51,334 | ,657 | -,38710 | ,86718 | -2,12775 | 1,35356 |
| Age_16_29_2017 | Assuming equal variances | 2,379 | ,127 | -,549 | 70 | ,585 | -,36111 | ,65806 | -1,67358 | ,95135 |
|  | No Assuming equal variances |  |  | -,549 | 58,979 | ,585 | -,36111 | ,65806 | -1,67790 | ,95568 |
| Age_16_29_2018 | Assuming equal variances | 3,668 | ,059 | -,950 | 74 | ,345 | -,47368 | ,49852 | -1,46701 | ,51964 |
|  | No Assuming equal variances |  |  | -,950 | 60,543 | ,346 | -,47368 | ,49852 | -1,47069 | ,52332 |
| Age_16_29_2019 | Assuming equal variances | 1,971 | ,164 | -,373 | 74 | ,710 | -,15789 | ,42353 | -1,00181 | ,68602 |
|  | No Assuming equal variances |  |  | -,373 | 60,262 | ,711 | -,15789 | ,42353 | -1,00501 | ,68922 |
| Age_16_29_2020 | Assuming equal variances | ,889 | ,349 | -,828 | 72 | ,410 | -,29730 | ,35906 | -1,01308 | ,41848 |
|  | No Assuming equal variances |  |  | -,828 | 63,154 | ,411 | -,29730 | ,35906 | -1,01479 | ,42020 |
| Age_16_29_2021 | Assuming equal variances | 1,189 | ,279 | ,105 | 68 | ,917 | ,02857 | ,27326 | -,51671 | ,57385 |
|  | No Assuming equal variances |  |  | ,105 | 64,559 | ,917 | ,02857 | ,27326 | -,51723 | ,57438 |
| Age_16_74_2015 | Assuming equal variances | 1,991 | ,163 | -1,268 | 62 | ,210 | -3,53125 | 2,78483 | -9,09804 | 2,03554 |
|  | No Assuming equal variances |  |  | -1,268 | 58,009 | ,210 | -3,53125 | 2,78483 | -9,10567 | 2,04317 |
| Age_16_74_2016 | Assuming equal variances | 1,214 | ,275 | -,986 | 60 | ,328 | -2,54839 | 2,58391 | -7,71698 | 2,62020 |
|  | No Assuming equal variances |  |  | -,986 | 56,702 | ,328 | -2,54839 | 2,58391 | -7,72317 | 2,62639 |
| Age_16_74_2017 | Assuming equal variances | 1,492 | ,226 | -1,080 | 70 | ,284 | -2,41667 | 2,23709 | -6,87840 | 2,04507 |
|  | No Assuming equal variances |  |  | -1,080 | 66,155 | ,284 | -2,41667 | 2,23709 | -6,88296 | 2,04963 |
| Age_16_74_2018 | Assuming equal variances | 1,206 | ,276 | -,914 | 74 | ,364 | -1,81579 | 1,98673 | -5,77443 | 2,14285 |
|  | No Assuming equal variances |  |  | -,914 | 70,905 | ,364 | -1,81579 | 1,98673 | -5,77730 | 2,14573 |
| Age_16_74_2019 | Assuming equal variances | 2,197 | ,143 | -1,016 | 74 | ,313 | -1,84211 | 1,81275 | -5,45409 | 1,76988 |
|  | No Assuming equal variances |  |  | -1,016 | 70,270 | ,313 | -1,84211 | 1,81275 | -5,45728 | 1,77307 |
| Age_16_74_2020 | Assuming equal variances | 3,651 | ,060 | -,930 | 74 | ,355 | -1,52632 | 1,64072 | -4,79552 | 1,74289 |
|  | No Assuming equal variances |  |  | -,930 | 68,951 | ,355 | -1,52632 | 1,64072 | -4,79950 | 1,74687 |
| Age_16_74_2021 | Assuming equal variances | 1,665 | ,201 | -1,011 | 72 | ,316 | -1,40541 | 1,39041 | -4,17714 | 1,36633 |
|  | No Assuming equal variances |  |  | -1,011 | 68,561 | ,316 | -1,40541 | 1,39041 | -4,17952 | 1,36871 |
| Age_20_24_2015 | Assuming equal variances | 1,106 | ,297 | -,584 | 62 | ,561 | -,62500 | 1,07045 | -2,76479 | 1,51479 |
|  | No Assuming equal variances |  |  | -,584 | 49,376 | ,562 | -,62500 | 1,07045 | -2,77573 | 1,52573 |
| Age_20_24_2016 | Assuming equal variances | ,609 | ,438 | -,474 | 60 | ,637 | -,38710 | ,81624 | -2,01982 | 1,24563 |
|  | No Assuming equal variances |  |  | -,474 | 49,872 | ,637 | -,38710 | ,81624 | -2,02667 | 1,25248 |
| Age_20_24_2017 | Assuming equal variances | 1,645 | ,204 | -,475 | 70 | ,636 | -,27778 | ,58460 | -1,44372 | ,88817 |
|  | No Assuming equal variances |  |  | -,475 | 63,707 | ,636 | -,27778 | ,58460 | -1,44575 | ,89019 |
| Age_20_24_2018 | Assuming equal variances | 3,371 | ,070 | -,803 | 72 | ,424 | -,35135 | ,43729 | -1,22306 | ,52036 |
|  | No Assuming equal variances |  |  | -,803 | 56,874 | ,425 | -,35135 | ,43729 | -1,22704 | ,52434 |
| Age_20_24_2019 | Assuming equal variances | ,011 | ,917 | ,333 | 74 | ,740 | ,13158 | ,39559 | -,65665 | ,91981 |
|  | No Assuming equal variances |  |  | ,333 | 69,180 | ,740 | ,13158 | ,39559 | -,65756 | ,92072 |
| Age_20_24_2020 | Assuming equal variances | 3,030 | ,086 | -,534 | 70 | ,595 | -,18610 | ,34825 | -,88067 | ,50847 |
|  | No Assuming equal variances |  |  | -,527 | 55,443 | ,600 | -,18610 | ,35282 | -,89304 | ,52084 |
| Age_20_24_2021 | Assuming equal variances | ,111 | ,740 | ,732 | 69 | ,467 | ,22778 | ,31118 | -,39301 | ,84856 |
|  | No Assuming equal variances |  |  | ,732 | 68,975 | ,466 | ,22778 | ,31097 | -,39259 | ,84814 |
| Age_25_29_2015 | Assuming equal variances | 1,262 | ,266 | -,817 | 60 | ,417 | -1,06452 | 1,30322 | -3,67133 | 1,54230 |
|  | No Assuming equal variances |  |  | -,817 | 50,214 | ,418 | -1,06452 | 1,30322 | -3,68182 | 1,55279 |
| Age_25_29_2016 | Assuming equal variances | ,615 | ,436 | -,398 | 59 | ,692 | -,41720 | 1,04783 | -2,51390 | 1,67949 |
|  | No Assuming equal variances |  |  | -,396 | 52,140 | ,694 | -,41720 | 1,05363 | -2,53133 | 1,69692 |
| Age_25_29_2017 | Assuming equal variances | 3,755 | ,057 | -,645 | 70 | ,521 | -,52778 | ,81826 | -2,15975 | 1,10420 |
|  | No Assuming equal variances |  |  | -,645 | 55,770 | ,522 | -,52778 | ,81826 | -2,16710 | 1,11155 |
| Age_25_29_2018 | Assuming equal variances | 2,383 | ,127 | -,910 | 74 | ,366 | -,52632 | ,57827 | -1,67854 | ,62591 |
|  | No Assuming equal variances |  |  | -,910 | 66,028 | ,366 | -,52632 | ,57827 | -1,68086 | ,62822 |
| Age_25_29_2019 | Assuming equal variances | 3,201 | ,078 | -,860 | 74 | ,393 | -,44737 | ,52025 | -1,48400 | ,58926 |
|  | No Assuming equal variances |  |  | -,860 | 62,544 | ,393 | -,44737 | ,52025 | -1,48716 | ,59242 |
| Age_25_29_2020 | Assuming equal variances | 3,075 | ,084 | -1,290 | 72 | ,201 | -,48649 | ,37725 | -1,23852 | ,26555 |
|  | No Assuming equal variances |  |  | -1,290 | 68,481 | ,202 | -,48649 | ,37725 | -1,23918 | ,26621 |
| Age_25_29_2021 | Assuming equal variances | ,728 | ,397 | -,470 | 68 | ,640 | -,17143 | ,36445 | -,89867 | ,55581 |
|  | No Assuming equal variances |  |  | -,470 | 57,978 | ,640 | -,17143 | ,36445 | -,90095 | ,55810 |
| Age_25_64_2015 | Assuming equal variances | 1,332 | ,253 | -,693 | 62 | ,491 | -1,90625 | 2,75208 | -7,40758 | 3,59508 |
|  | No Assuming equal variances |  |  | -,693 | 58,003 | ,491 | -1,90625 | 2,75208 | -7,41513 | 3,60263 |
| Age_25_64_2016 | Assuming equal variances | ,454 | ,503 | -,335 | 60 | ,739 | -,83871 | 2,50440 | -5,84825 | 4,17083 |
|  | No Assuming equal variances |  |  | -,335 | 57,058 | ,739 | -,83871 | 2,50440 | -5,85357 | 4,17615 |
| Age_25_64_2017 | Assuming equal variances | ,849 | ,360 | -,386 | 70 | ,701 | -,77778 | 2,01628 | -4,79912 | 3,24357 |
|  | No Assuming equal variances |  |  | -,386 | 65,745 | ,701 | -,77778 | 2,01628 | -4,80370 | 3,24815 |
| Age_25_64_2018 | Assuming equal variances | 1,468 | ,230 | -,315 | 74 | ,754 | -,57895 | 1,83768 | -4,24060 | 3,08271 |
|  | No Assuming equal variances |  |  | -,315 | 69,533 | ,754 | -,57895 | 1,83768 | -4,24452 | 3,08662 |
| Age_25_64_2019 | Assuming equal variances | ,909 | ,343 | -,201 | 74 | ,841 | -,31579 | 1,57022 | -3,44453 | 2,81295 |
|  | No Assuming equal variances |  |  | -,201 | 71,226 | ,841 | -,31579 | 1,57022 | -3,44655 | 2,81497 |
| Age_25_64_2020 | Assuming equal variances | 2,086 | ,153 | -,371 | 74 | ,711 | -,47368 | 1,27581 | -3,01579 | 2,06842 |
|  | No Assuming equal variances |  |  | -,371 | 68,568 | ,712 | -,47368 | 1,27581 | -3,01914 | 2,07177 |
| Age_25_64_2021 | Assuming equal variances | 1,125 | ,293 | -,203 | 70 | ,839 | -,22222 | 1,09262 | -2,40139 | 1,95695 |
|  | No Assuming equal variances |  |  | -,203 | 66,011 | ,839 | -,22222 | 1,09262 | -2,40371 | 1,95927 |
| Age_55_74 | Assuming equal variances | ,524 | ,472 | -1,180 | 62 | ,243 | -6,46875 | 5,48240 | -17,42790 | 4,49040 |
|  | No Assuming equal variances |  |  | -1,180 | 61,093 | ,243 | -6,46875 | 5,48240 | -17,43114 | 4,49364 |
| Age_55_75 | Assuming equal variances | ,568 | ,454 | -,865 | 60 | ,391 | -4,70968 | 5,44701 | -15,60532 | 6,18597 |
|  | No Assuming equal variances |  |  | -,865 | 58,905 | ,391 | -4,70968 | 5,44701 | -15,60949 | 6,19013 |
| Age_55_76 | Assuming equal variances | ,713 | ,401 | -1,010 | 70 | ,316 | -4,77778 | 4,72889 | -14,20925 | 4,65369 |
|  | No Assuming equal variances |  |  | -1,010 | 68,674 | ,316 | -4,77778 | 4,72889 | -14,21245 | 4,65690 |
| Age_55_77 | Assuming equal variances | ,201 | ,655 | -,634 | 74 | ,528 | -2,73684 | 4,31640 | -11,33746 | 5,86377 |
|  | No Assuming equal variances |  |  | -,634 | 73,375 | ,528 | -2,73684 | 4,31640 | -11,33868 | 5,86499 |
| Age_55_78 | Assuming equal variances | 1,216 | ,274 | -,807 | 74 | ,422 | -3,21053 | 3,98047 | -11,14179 | 4,72074 |
|  | No Assuming equal variances |  |  | -,807 | 72,384 | ,423 | -3,21053 | 3,98047 | -11,14473 | 4,72368 |
| Age_55_79 | Assuming equal variances | 1,745 | ,191 | -,781 | 74 | ,437 | -2,94737 | 3,77524 | -10,46969 | 4,57496 |
|  | No Assuming equal variances |  |  | -,781 | 71,373 | ,438 | -2,94737 | 3,77524 | -10,47429 | 4,57956 |
| Age_55_80 | Assuming equal variances | 1,410 | ,239 | -,796 | 70 | ,429 | -2,66667 | 3,34898 | -9,34601 | 4,01267 |
|  | No Assuming equal variances |  |  | -,796 | 67,602 | ,429 | -2,66667 | 3,34898 | -9,35017 | 4,01684 |

### Stage 1: never access to the Internet / Variable: Education

| **Test for independent samples** | | | | | | | | | | |
| --- | --- | --- | --- | --- | --- | --- | --- | --- | --- | --- |
|  | | Levene’s test of equality of variances | | t test for mean equality | | | | | | |
|  |  | F | Sig. | t | df | Sig. (two-tailed) | Means difference | Difference standard error | 95% confidence interval of the difference | |
|  |  |  |  |  |  |  |  |  | Lower | Upper |
| Low Formal Education_2015 | Assuming equal variances | ,434 | ,512 | -1,496 | 74 | ,139 | -6,89474 | 4,60854 | -16,07744 | 2,28797 |
|  | No Assuming equal variances |  |  | -1,496 | 73,029 | ,139 | -6,89474 | 4,60854 | -16,07947 | 2,29000 |
| Low Formal Education_2016 | Assuming equal variances | ,407 | ,525 | -1,378 | 74 | ,172 | -6,13158 | 4,44891 | -14,99622 | 2,73306 |
|  | No Assuming equal variances |  |  | -1,378 | 73,209 | ,172 | -6,13158 | 4,44891 | -14,99781 | 2,73466 |
| Low Formal Education_2017 | Assuming equal variances | ,544 | ,463 | -1,626 | 74 | ,108 | -7,23684 | 4,45140 | -16,10645 | 1,63276 |
|  | No Assuming equal variances |  |  | -1,626 | 72,191 | ,108 | -7,23684 | 4,45140 | -16,11014 | 1,63646 |
| Low Formal Education_2018 | Assuming equal variances | ,752 | ,389 | -1,391 | 74 | ,168 | -5,60526 | 4,02914 | -13,63350 | 2,42298 |
|  | No Assuming equal variances |  |  | -1,391 | 73,731 | ,168 | -5,60526 | 4,02914 | -13,63399 | 2,42346 |
| Low Formal Education_2019 | Assuming equal variances | 1,191 | ,279 | -1,756 | 74 | ,083 | -6,65789 | 3,79156 | -14,21274 | ,89695 |
|  | No Assuming equal variances |  |  | -1,756 | 73,136 | ,083 | -6,65789 | 3,79156 | -14,21423 | ,89844 |
| Low Formal Education_2020 | Assuming equal variances | 1,169 | ,283 | -1,342 | 74 | ,184 | -4,78947 | 3,56985 | -11,90256 | 2,32361 |
|  | No Assuming equal variances |  |  | -1,342 | 71,375 | ,184 | -4,78947 | 3,56985 | -11,90691 | 2,32796 |
| Low Formal Education_2021 | Assuming equal variances | 1,547 | ,218 | -1,409 | 74 | ,163 | -4,39474 | 3,11999 | -10,61145 | 1,82197 |
|  | No Assuming equal variances |  |  | -1,409 | 71,315 | ,163 | -4,39474 | 3,11999 | -10,61534 | 1,82586 |
| Medium Formal Education_2015 | Assuming equal variances | ,853 | ,359 | -,658 | 74 | ,512 | -1,44737 | 2,19830 | -5,82758 | 2,93285 |
|  | No Assuming equal variances |  |  | -,658 | 72,809 | ,512 | -1,44737 | 2,19830 | -5,82878 | 2,93404 |
| Medium Formal Education_2016 | Assuming equal variances | ,127 | ,722 | -,383 | 73 | ,703 | -,80014 | 2,08724 | -4,96001 | 3,35972 |
|  | No Assuming equal variances |  |  | -,384 | 72,531 | ,702 | -,80014 | 2,08422 | -4,95443 | 3,35415 |
| Medium Formal Education_2017 | Assuming equal variances | ,270 | ,605 | -,320 | 74 | ,750 | -,63158 | 1,97625 | -4,56934 | 3,30618 |
|  | No Assuming equal variances |  |  | -,320 | 72,643 | ,750 | -,63158 | 1,97625 | -4,57056 | 3,30740 |
| Medium Formal Education_2018 | Assuming equal variances | ,003 | ,955 | ,016 | 74 | ,987 | ,02632 | 1,63122 | -3,22396 | 3,27659 |
|  | No Assuming equal variances |  |  | ,016 | 73,734 | ,987 | ,02632 | 1,63122 | -3,22415 | 3,27678 |
| Medium Formal Education_2019 | Assuming equal variances | ,401 | ,528 | ,017 | 74 | ,986 | ,02632 | 1,53773 | -3,03768 | 3,09031 |
|  | No Assuming equal variances |  |  | ,017 | 73,394 | ,986 | ,02632 | 1,53773 | -3,03810 | 3,09074 |
| Medium Formal Education_2020 | Assuming equal variances | ,259 | ,613 | -,348 | 73 | ,729 | -,48578 | 1,39422 | -3,26445 | 2,29290 |
|  | No Assuming equal variances |  |  | -,348 | 72,546 | ,729 | -,48578 | 1,39520 | -3,26670 | 2,29515 |
| Medium Formal Education_2021 | Assuming equal variances | ,381 | ,539 | ,095 | 72 | ,925 | ,10380 | 1,09410 | -2,07725 | 2,28485 |
|  | No Assuming equal variances |  |  | ,095 | 71,777 | ,924 | ,10380 | 1,09080 | -2,07078 | 2,27838 |
| High Formal Education_2015 | Assuming equal variances | ,774 | ,382 | ,437 | 68 | ,663 | ,24079 | ,55076 | -,85823 | 1,33981 |
|  | No Assuming equal variances |  |  | ,432 | 61,349 | ,667 | ,24079 | ,55770 | -,87428 | 1,35585 |
| High Formal Education_2016 | Assuming equal variances | ,171 | ,681 | ,392 | 67 | ,696 | ,17061 | ,43489 | -,69744 | 1,03866 |
|  | No Assuming equal variances |  |  | ,393 | 66,014 | ,696 | ,17061 | ,43408 | -,69607 | 1,03728 |
| High Formal Education_2017 | Assuming equal variances | ,503 | ,480 | ,201 | 72 | ,841 | ,11988 | ,59693 | -1,07007 | 1,30984 |
|  | No Assuming equal variances |  |  | ,200 | 69,659 | ,842 | ,11988 | ,59904 | -1,07497 | 1,31474 |
| High Formal Education_2018 | Assuming equal variances | 2,566 | ,113 | 1,094 | 74 | ,278 | ,55263 | ,50529 | -,45418 | 1,55944 |
|  | No Assuming equal variances |  |  | 1,094 | 65,208 | ,278 | ,55263 | ,50529 | -,45644 | 1,56170 |
| High Formal Education_2019 | Assuming equal variances | ,249 | ,619 | ,613 | 74 | ,542 | ,21053 | ,34328 | -,47347 | ,89453 |
|  | No Assuming equal variances |  |  | ,613 | 73,691 | ,542 | ,21053 | ,34328 | -,47352 | ,89457 |
| High Formal Education_2020 | Assuming equal variances | ,361 | ,550 | ,721 | 73 | ,473 | ,23471 | ,32547 | -,41395 | ,88337 |
|  | No Assuming equal variances |  |  | ,723 | 70,047 | ,472 | ,23471 | ,32445 | -,41239 | ,88180 |
| High Formal Education_2021 | Assuming equal variances | 1,557 | ,216 | 1,145 | 72 | ,256 | ,29971 | ,26179 | -,22215 | ,82157 |
|  | No Assuming equal variances |  |  | 1,137 | 66,080 | ,260 | ,29971 | ,26356 | -,22649 | ,82591 |

### Stages 2 and 3 access once a week to the Internet / variable: age

| **Test for independent samples** | | | | | | | | | | |
| --- | --- | --- | --- | --- | --- | --- | --- | --- | --- | --- |
|  | | Levene’s test of equality of variances | | t test for mean equality | | | | | | |
|  |  | F | Sig. | t | df | Sig. (two-tailed) | Means difference | Difference standard error | 95% confidence interval of the difference | |
|  |  |  |  |  |  |  |  |  | Lower | Upper |
| Used_the_Internet_once_week_Age_16_19_2015 | Assuming equal variances | ,856 | ,359 | ,193 | 58 | ,848 | ,30000 | 1,55671 | -2,81609 | 3,41609 |
|  | No Assuming equal variances |  |  | ,193 | 51,263 | ,848 | ,30000 | 1,55671 | -2,82483 | 3,42483 |
| Used_the_Internet_once_week_Age_16_19_2016 | Assuming equal variances | 1,406 | ,241 | ,493 | 58 | ,624 | ,66667 | 1,35092 | -2,03749 | 3,37082 |
|  | No Assuming equal variances |  |  | ,493 | 52,381 | ,624 | ,66667 | 1,35092 | -2,04368 | 3,37701 |
| Used_the_Internet_once_week_Age_16_19_2017 | Assuming equal variances | ,191 | ,663 | ,107 | 68 | ,915 | ,11275 | 1,05731 | -1,99708 | 2,22257 |
|  | No Assuming equal variances |  |  | ,106 | 62,164 | ,916 | ,11275 | 1,06501 | -2,01607 | 2,24156 |
| Used_the_Internet_once_week_Age_16_19_2018 | Assuming equal variances | 2,964 | ,090 | 1,532 | 69 | ,130 | 1,47143 | ,96062 | -,44496 | 3,38782 |
|  | No Assuming equal variances |  |  | 1,522 | 57,291 | ,133 | 1,47143 | ,96649 | -,46372 | 3,40658 |
| Used_the_Internet_once_week_Age_16_19_2019 | Assuming equal variances | 5,773 | ,019 | ,854 | 71 | ,396 | ,70721 | ,82800 | -,94379 | 2,35820 |
|  | No Assuming equal variances |  |  | ,860 | 59,303 | ,393 | ,70721 | ,82263 | -,93869 | 2,35311 |
| Used_the_Internet_once_week_Age_16_19_2020 | Assuming equal variances | ,428 | ,515 | ,121 | 62 | ,904 | ,09873 | ,81759 | -1,53560 | 1,73306 |
|  | No Assuming equal variances |  |  | ,120 | 56,559 | ,905 | ,09873 | ,82407 | -1,55172 | 1,74918 |
| Used_the_Internet_once_week_Age_16_19_2021 | Assuming equal variances | 4,323 | ,041 | ,730 | 67 | ,468 | ,38739 | ,53063 | -,67175 | 1,44654 |
|  | No Assuming equal variances |  |  | ,733 | 62,131 | ,466 | ,38739 | ,52823 | -,66848 | 1,44327 |
| Used_the_Internet_once_week_Age_16_24_2015 | Assuming equal variances | ,563 | ,456 | ,021 | 62 | ,983 | ,03125 | 1,49637 | -2,95996 | 3,02246 |
|  | No Assuming equal variances |  |  | ,021 | 54,471 | ,983 | ,03125 | 1,49637 | -2,96820 | 3,03070 |
| Used_the_Internet_once_week_Age_16_24_2016 | Assuming equal variances | ,662 | ,419 | ,551 | 60 | ,584 | ,67742 | 1,22917 | -1,78128 | 3,13612 |
|  | No Assuming equal variances |  |  | ,551 | 53,406 | ,584 | ,67742 | 1,22917 | -1,78754 | 3,14238 |
| Used_the_Internet_once_week_Age_16_24_2017 | Assuming equal variances | ,856 | ,358 | ,275 | 70 | ,784 | ,25000 | ,90933 | -1,56359 | 2,06359 |
|  | No Assuming equal variances |  |  | ,275 | 64,416 | ,784 | ,25000 | ,90933 | -1,56636 | 2,06636 |
| Used_the_Internet_once_week_Age_16_24_2018 | Assuming equal variances | 2,087 | ,153 | ,828 | 72 | ,410 | ,62162 | ,75078 | -,87503 | 2,11828 |
|  | No Assuming equal variances |  |  | ,828 | 60,913 | ,411 | ,62162 | ,75078 | -,87970 | 2,12294 |
| Used_the_Internet_once_week_Age_16_24_2019 | Assuming equal variances | 3,135 | ,081 | ,639 | 74 | ,525 | ,42105 | ,65888 | -,89179 | 1,73389 |
|  | No Assuming equal variances |  |  | ,639 | 65,187 | ,525 | ,42105 | ,65888 | -,89474 | 1,73685 |
| Used_the_Internet_once_week_Age_16_24_2020 | Assuming equal variances | ,140 | ,709 | ,138 | 68 | ,891 | ,08571 | ,62116 | -1,15378 | 1,32521 |
|  | No Assuming equal variances |  |  | ,138 | 65,999 | ,891 | ,08571 | ,62116 | -1,15446 | 1,32589 |
| Used_the_Internet_once_week_Age_16_24_2021 | Assuming equal variances | ,036 | ,851 | -,489 | 68 | ,627 | -,22857 | ,46768 | -1,16182 | ,70468 |
|  | No Assuming equal variances |  |  | -,489 | 67,781 | ,627 | -,22857 | ,46768 | -1,16187 | ,70473 |
| Used_the_Internet_once_week_Age_16_29_2015 | Assuming equal variances | ,857 | ,358 | ,251 | 62 | ,802 | ,40625 | 1,61620 | -2,82450 | 3,63700 |
|  | No Assuming equal variances |  |  | ,251 | 55,547 | ,802 | ,40625 | 1,61620 | -2,83198 | 3,64448 |
| Used_the_Internet_once_week_Age_16_29_2016 | Assuming equal variances | ,820 | ,369 | ,150 | 60 | ,881 | ,19355 | 1,28720 | -2,38124 | 2,76833 |
|  | No Assuming equal variances |  |  | ,150 | 54,575 | ,881 | ,19355 | 1,28720 | -2,38651 | 2,77361 |
| Used_the_Internet_once_week_Age_16_29_2017 | Assuming equal variances | 2,119 | ,150 | ,533 | 70 | ,596 | ,52778 | ,98975 | -1,44622 | 2,50177 |
|  | No Assuming equal variances |  |  | ,533 | 63,080 | ,596 | ,52778 | ,98975 | -1,45003 | 2,50559 |
| Used_the_Internet_once_week_Age_16_29_2018 | Assuming equal variances | 2,154 | ,147 | ,611 | 72 | ,543 | ,48649 | ,79660 | -1,10150 | 2,07447 |
|  | No Assuming equal variances |  |  | ,611 | 66,905 | ,543 | ,48649 | ,79660 | -1,10357 | 2,07654 |
| Used_the_Internet_once_week_Age_16_29_2019 | Assuming equal variances | 4,254 | ,043 | ,873 | 74 | ,385 | ,60526 | ,69330 | -,77617 | 1,98669 |
|  | No Assuming equal variances |  |  | ,873 | 66,070 | ,386 | ,60526 | ,69330 | -,77893 | 1,98945 |
| Used_the_Internet_once_week_Age_16_29_2020 | Assuming equal variances | ,007 | ,935 | ,400 | 68 | ,690 | ,25714 | ,64281 | -1,02557 | 1,53985 |
|  | No Assuming equal variances |  |  | ,400 | 66,928 | ,690 | ,25714 | ,64281 | -1,02594 | 1,54022 |
| Used_the_Internet_once_week_Age_16_29_2021 | Assuming equal variances | ,084 | ,772 | -,244 | 68 | ,808 | -,11429 | ,46856 | -1,04927 | ,82070 |
|  | No Assuming equal variances |  |  | -,244 | 67,989 | ,808 | -,11429 | ,46856 | -1,04928 | ,82070 |
| Used_the_Internet_once_week_Age_16_74_2015 | Assuming equal variances | ,743 | ,392 | 1,128 | 62 | ,264 | 3,62500 | 3,21463 | -2,80096 | 10,05096 |
|  | No Assuming equal variances |  |  | 1,128 | 60,166 | ,264 | 3,62500 | 3,21463 | -2,80486 | 10,05486 |
| Used_the_Internet_once_week_Age_16_74_2016 | Assuming equal variances | ,434 | ,512 | ,909 | 60 | ,367 | 2,67742 | 2,94609 | -3,21563 | 8,57047 |
|  | No Assuming equal variances |  |  | ,909 | 58,348 | ,367 | 2,67742 | 2,94609 | -3,21907 | 8,57391 |
| Used_the_Internet_once_week_Age_16_74_2017 | Assuming equal variances | ,843 | ,362 | 1,033 | 70 | ,305 | 2,69444 | 2,60950 | -2,51004 | 7,89893 |
|  | No Assuming equal variances |  |  | 1,033 | 67,360 | ,306 | 2,69444 | 2,60950 | -2,51363 | 7,90252 |
| Used_the_Internet_once_week_Age_16_74_2018 | Assuming equal variances | 1,271 | ,263 | ,824 | 72 | ,412 | 1,91892 | 2,32795 | -2,72177 | 6,55961 |
|  | No Assuming equal variances |  |  | ,824 | 69,851 | ,413 | 1,91892 | 2,32795 | -2,72421 | 6,56205 |
| Used_the_Internet_once_week_Age_16_74_2019 | Assuming equal variances | 1,476 | ,228 | 1,060 | 74 | ,292 | 2,26316 | 2,13458 | -1,99008 | 6,51639 |
|  | No Assuming equal variances |  |  | 1,060 | 71,954 | ,293 | 2,26316 | 2,13458 | -1,99209 | 6,51840 |
| Used_the_Internet_once_week_Age_16_74_2020 | Assuming equal variances | 2,743 | ,102 | ,860 | 70 | ,393 | 1,72222 | 2,00369 | -2,27401 | 5,71845 |
|  | No Assuming equal variances |  |  | ,860 | 67,066 | ,393 | 1,72222 | 2,00369 | -2,27709 | 5,72153 |
| Used_the_Internet_once_week_Age_16_74_2021 | Assuming equal variances | 1,758 | ,189 | ,742 | 70 | ,461 | 1,25000 | 1,68510 | -2,11083 | 4,61083 |
|  | No Assuming equal variances |  |  | ,742 | 67,167 | ,461 | 1,25000 | 1,68510 | -2,11333 | 4,61333 |
| Used_the_Internet_once_week_Age_20_24_2015 | Assuming equal variances | ,286 | ,595 | ,138 | 62 | ,890 | ,21875 | 1,58224 | -2,94411 | 3,38161 |
|  | No Assuming equal variances |  |  | ,138 | 56,217 | ,891 | ,21875 | 1,58224 | -2,95060 | 3,38810 |
| Used_the_Internet_once_week_Age_20_24_2016 | Assuming equal variances | ,577 | ,450 | ,316 | 60 | ,753 | ,38710 | 1,22326 | -2,05978 | 2,83398 |
|  | No Assuming equal variances |  |  | ,316 | 53,979 | ,753 | ,38710 | 1,22326 | -2,06541 | 2,83960 |
| Used_the_Internet_once_week_Age_20_24_2017 | Assuming equal variances | 2,499 | ,118 | ,479 | 70 | ,633 | ,44444 | ,92741 | -1,40522 | 2,29411 |
|  | No Assuming equal variances |  |  | ,479 | 63,259 | ,633 | ,44444 | ,92741 | -1,40870 | 2,29759 |
| Used_the_Internet_once_week_Age_20_24_2018 | Assuming equal variances | 5,248 | ,025 | ,451 | 72 | ,653 | ,32432 | ,71863 | -1,10825 | 1,75690 |
|  | No Assuming equal variances |  |  | ,451 | 60,124 | ,653 | ,32432 | ,71863 | -1,11310 | 1,76175 |
| Used_the_Internet_once_week_Age_20_24_2019 | Assuming equal variances | ,277 | ,600 | ,319 | 74 | ,751 | ,21053 | ,65990 | -1,10435 | 1,52540 |
|  | No Assuming equal variances |  |  | ,319 | 71,982 | ,751 | ,21053 | ,65990 | -1,10496 | 1,52601 |
| Used_the_Internet_once_week_Age_20_24_2020 | Assuming equal variances | ,633 | ,429 | ,369 | 68 | ,713 | ,22857 | ,61961 | -1,00784 | 1,46498 |
|  | No Assuming equal variances |  |  | ,369 | 67,405 | ,713 | ,22857 | ,61961 | -1,00803 | 1,46518 |
| Used_the_Internet_once_week_Age_20_24_2021 | Assuming equal variances | ,559 | ,457 | -1,347 | 68 | ,183 | -,74286 | ,55163 | -1,84362 | ,35791 |
|  | No Assuming equal variances |  |  | -1,347 | 67,298 | ,183 | -,74286 | ,55163 | -1,84383 | ,35811 |
| Used_the_Internet_once_week_Age_25_29_2015 | Assuming equal variances | 1,296 | ,259 | ,504 | 60 | ,616 | 1,00000 | 1,98312 | -2,96684 | 4,96684 |
|  | No Assuming equal variances |  |  | ,504 | 54,940 | ,616 | 1,00000 | 1,98312 | -2,97437 | 4,97437 |
| Used_the_Internet_once_week_Age_25_29_2016 | Assuming equal variances | ,131 | ,718 | -,233 | 58 | ,817 | -,36667 | 1,57428 | -3,51793 | 2,78459 |
|  | No Assuming equal variances |  |  | -,233 | 54,736 | ,817 | -,36667 | 1,57428 | -3,52193 | 2,78860 |
| Used_the_Internet_once_week_Age_25_29_2017 | Assuming equal variances | 3,001 | ,088 | ,760 | 70 | ,450 | ,91667 | 1,20572 | -1,48807 | 3,32141 |
|  | No Assuming equal variances |  |  | ,760 | 62,347 | ,450 | ,91667 | 1,20572 | -1,49327 | 3,32661 |
| Used_the_Internet_once_week_Age_25_29_2018 | Assuming equal variances | 1,606 | ,209 | ,651 | 72 | ,517 | ,62162 | ,95436 | -1,28086 | 2,52410 |
|  | No Assuming equal variances |  |  | ,651 | 71,662 | ,517 | ,62162 | ,95436 | -1,28101 | 2,52426 |
| Used_the_Internet_once_week_Age_25_29_2019 | Assuming equal variances | 1,806 | ,183 | ,910 | 74 | ,366 | ,76316 | ,83842 | -,90743 | 2,43374 |
|  | No Assuming equal variances |  |  | ,910 | 70,060 | ,366 | ,76316 | ,83842 | -,90899 | 2,43531 |
| Used_the_Internet_once_week_Age_25_29_2020 | Assuming equal variances | ,106 | ,746 | ,508 | 67 | ,613 | ,41765 | ,82141 | -1,22189 | 2,05718 |
|  | No Assuming equal variances |  |  | ,508 | 66,942 | ,613 | ,41765 | ,82141 | -1,22191 | 2,05721 |
| Used_the_Internet_once_week_Age_25_29_2021 | Assuming equal variances | ,418 | ,520 | -,050 | 68 | ,960 | -,02857 | ,56899 | -1,16397 | 1,10682 |
|  | No Assuming equal variances |  |  | -,050 | 66,815 | ,960 | -,02857 | ,56899 | -1,16433 | 1,10719 |
| Used_the_Internet_once_week_Age_25_64_2015 | Assuming equal variances | ,532 | ,468 | ,590 | 62 | ,557 | 1,96875 | 3,33433 | -4,69649 | 8,63399 |
|  | No Assuming equal variances |  |  | ,590 | 60,269 | ,557 | 1,96875 | 3,33433 | -4,70030 | 8,63780 |
| Used_the_Internet_once_week_Age_25_64_2016 | Assuming equal variances | ,361 | ,550 | ,263 | 60 | ,794 | ,77419 | 2,94765 | -5,12199 | 6,67038 |
|  | No Assuming equal variances |  |  | ,263 | 58,382 | ,794 | ,77419 | 2,94765 | -5,12535 | 6,67374 |
| Used_the_Internet_once_week_Age_25_64_2017 | Assuming equal variances | ,511 | ,477 | ,323 | 70 | ,748 | ,80556 | 2,49756 | -4,17567 | 5,78678 |
|  | No Assuming equal variances |  |  | ,323 | 67,577 | ,748 | ,80556 | 2,49756 | -4,17881 | 5,78993 |
| Used_the_Internet_once_week_Age_25_64_2018 | Assuming equal variances | ,937 | ,336 | ,110 | 72 | ,913 | ,24324 | 2,20859 | -4,15951 | 4,64600 |
|  | No Assuming equal variances |  |  | ,110 | 69,627 | ,913 | ,24324 | 2,20859 | -4,16208 | 4,64856 |
| Used_the_Internet_once_week_Age_25_64_2019 | Assuming equal variances | ,931 | ,338 | ,217 | 74 | ,829 | ,42105 | 1,93916 | -3,44281 | 4,28492 |
|  | No Assuming equal variances |  |  | ,217 | 72,359 | ,829 | ,42105 | 1,93916 | -3,44427 | 4,28637 |
| Used_the_Internet_once_week_Age_25_64_2020 | Assuming equal variances | 2,316 | ,133 | ,275 | 70 | ,784 | ,47222 | 1,72017 | -2,95854 | 3,90299 |
|  | No Assuming equal variances |  |  | ,275 | 67,780 | ,785 | ,47222 | 1,72017 | -2,96052 | 3,90496 |
| Used_the_Internet_once_week_Age_25_64_2021 | Assuming equal variances | 2,692 | ,105 | -,084 | 70 | ,934 | -,11111 | 1,33029 | -2,76429 | 2,54206 |
|  | No Assuming equal variances |  |  | -,084 | 66,666 | ,934 | -,11111 | 1,33029 | -2,76662 | 2,54440 |
| Used_the_Internet_once_week_Age_55_74 | Assuming equal variances | ,024 | ,877 | 1,308 | 62 | ,196 | 7,12500 | 5,44564 | -3,76068 | 18,01068 |
|  | No Assuming equal variances |  |  | 1,308 | 61,934 | ,196 | 7,12500 | 5,44564 | -3,76091 | 18,01091 |
| Used_the_Internet_once_week_Age_55_75 | Assuming equal variances | ,041 | ,839 | ,944 | 60 | ,349 | 5,16129 | 5,46892 | -5,77819 | 16,10077 |
|  | No Assuming equal variances |  |  | ,944 | 59,734 | ,349 | 5,16129 | 5,46892 | -5,77919 | 16,10177 |
| Used_the_Internet_once_week_Age_55_76 | Assuming equal variances | ,507 | ,479 | 1,000 | 70 | ,321 | 5,00000 | 4,99818 | -4,96856 | 14,96856 |
|  | No Assuming equal variances |  |  | 1,000 | 69,239 | ,321 | 5,00000 | 4,99818 | -4,97049 | 14,97049 |
| Used_the_Internet_once_week_Age_55_77 | Assuming equal variances | ,185 | ,668 | ,624 | 72 | ,535 | 2,86486 | 4,59030 | -6,28572 | 12,01545 |
|  | No Assuming equal variances |  |  | ,624 | 71,524 | ,535 | 2,86486 | 4,59030 | -6,28676 | 12,01649 |
| Used_the_Internet_once_week_Age_55_78 | Assuming equal variances | ,521 | ,473 | ,940 | 74 | ,350 | 3,97368 | 4,22704 | -4,44888 | 12,39625 |
|  | No Assuming equal variances |  |  | ,940 | 73,385 | ,350 | 3,97368 | 4,22704 | -4,45005 | 12,39742 |
| Used_the_Internet_once_week_Age_55_79 | Assuming equal variances | 1,093 | ,300 | ,717 | 70 | ,476 | 3,00000 | 4,18644 | -5,34959 | 11,34959 |
|  | No Assuming equal variances |  |  | ,717 | 68,828 | ,476 | 3,00000 | 4,18644 | -5,35209 | 11,35209 |
| Used_the_Internet_once_week_Age_55_80 | Assuming equal variances | ,785 | ,379 | ,599 | 70 | ,551 | 2,25000 | 3,75769 | -5,24448 | 9,74448 |
|  | No Assuming equal variances |  |  | ,599 | 68,888 | ,551 | 2,25000 | 3,75769 | -5,24661 | 9,74661 |

### Stages 2 and 3 access daily to the Internet / variable: education

| **Test for independent samples** | | | | | | | | | | |
| --- | --- | --- | --- | --- | --- | --- | --- | --- | --- | --- |
|  | | Levene’s test of equality of variances | | t test for mean equality | | | | | | |
|  |  | F | Sig. | t | df | Sig. (two-tailed) | Means difference | Difference standard error | 95% confidence interval of the difference | |
|  |  |  |  |  |  |  |  |  | Lower | Upper |
| Used_the_Internet_once_week_LowFormalEducation_2015 | Assuming equal variances | ,064 | ,802 | 1,634 | 60 | ,107 | 7,93548 | 4,85641 | -1,77879 | 17,64975 |
|  | No Assuming equal variances |  |  | 1,634 | 59,511 | ,108 | 7,93548 | 4,85641 | -1,78043 | 17,65140 |
| Used_the_Internet_once_week_LowFormalEducation_2016 | Assuming equal variances | ,233 | ,631 | 1,597 | 60 | ,115 | 7,58065 | 4,74553 | -1,91182 | 17,07311 |
|  | No Assuming equal variances |  |  | 1,597 | 59,361 | ,115 | 7,58065 | 4,74553 | -1,91392 | 17,07521 |
| Used_the_Internet_once_week_LowFormalEducation_2017 | Assuming equal variances | 1,609 | ,209 | 1,673 | 68 | ,099 | 7,68571 | 4,59496 | -1,48339 | 16,85482 |
|  | No Assuming equal variances |  |  | 1,673 | 64,614 | ,099 | 7,68571 | 4,59496 | -1,49209 | 16,86352 |
| Used_the_Internet_once_week_LowFormalEducation_2018 | Assuming equal variances | 1,134 | ,290 | 1,368 | 72 | ,176 | 5,72973 | 4,18875 | -2,62040 | 14,07986 |
|  | No Assuming equal variances |  |  | 1,368 | 71,474 | ,176 | 5,72973 | 4,18875 | -2,62145 | 14,08091 |
| Used_the_Internet_once_week_LowFormalEducation_2019 | Assuming equal variances | ,908 | ,344 | 1,726 | 74 | ,089 | 6,84211 | 3,96511 | -1,05856 | 14,74277 |
|  | No Assuming equal variances |  |  | 1,726 | 73,231 | ,089 | 6,84211 | 3,96511 | -1,05994 | 14,74415 |
| Used_the_Internet_once_week_LowFormalEducation_2020 | Assuming equal variances | ,458 | ,501 | 1,271 | 68 | ,208 | 5,22857 | 4,11310 | -2,97898 | 13,43612 |
|  | No Assuming equal variances |  |  | 1,271 | 66,627 | ,208 | 5,22857 | 4,11310 | -2,98204 | 13,43919 |
| Used_the_Internet_once_week_LowFormalEducation_2021 | Assuming equal variances | 1,805 | ,183 | 1,360 | 70 | ,178 | 4,72222 | 3,47238 | -2,20323 | 11,64767 |
|  | No Assuming equal variances |  |  | 1,360 | 67,583 | ,178 | 4,72222 | 3,47238 | -2,20759 | 11,65203 |
| Used_the_Internet_once_week_MediumFormalEducation_2015 | Assuming equal variances | ,186 | ,668 | ,768 | 60 | ,446 | 2,38710 | 3,11001 | -3,83384 | 8,60804 |
|  | No Assuming equal variances |  |  | ,768 | 59,750 | ,446 | 2,38710 | 3,11001 | -3,83438 | 8,60857 |
| Used_the_Internet_once_week_MediumFormalEducation_2016 | Assuming equal variances | ,163 | ,688 | ,533 | 60 | ,596 | 1,51613 | 2,84461 | -4,17393 | 7,20619 |
|  | No Assuming equal variances |  |  | ,533 | 59,658 | ,596 | 1,51613 | 2,84461 | -4,17460 | 7,20686 |
| Used_the_Internet_once_week_MediumFormalEducation_2017 | Assuming equal variances | ,426 | ,516 | ,513 | 70 | ,610 | 1,33333 | 2,60127 | -3,85474 | 6,52141 |
|  | No Assuming equal variances |  |  | ,513 | 69,417 | ,610 | 1,33333 | 2,60127 | -3,85551 | 6,52218 |
| Used_the_Internet_once_week_MediumFormalEducation_2018 | Assuming equal variances | ,054 | ,817 | ,049 | 72 | ,961 | ,10811 | 2,20567 | -4,28882 | 4,50503 |
|  | No Assuming equal variances |  |  | ,049 | 71,781 | ,961 | ,10811 | 2,20567 | -4,28905 | 4,50526 |
| Used_the_Internet_once_week_MediumFormalEducation_2019 | Assuming equal variances | ,531 | ,468 | ,395 | 74 | ,694 | ,78947 | 1,99905 | -3,19373 | 4,77268 |
|  | No Assuming equal variances |  |  | ,395 | 73,497 | ,694 | ,78947 | 1,99905 | -3,19418 | 4,77313 |
| Used_the_Internet_once_week_MediumFormalEducation_2020 | Assuming equal variances | ,572 | ,452 | ,207 | 70 | ,837 | ,38889 | 1,87938 | -3,35941 | 4,13719 |
|  | No Assuming equal variances |  |  | ,207 | 69,512 | ,837 | ,38889 | 1,87938 | -3,35987 | 4,13765 |
| Used_the_Internet_once_week_MediumFormalEducation_2021 | Assuming equal variances | 1,010 | ,318 | ,149 | 70 | ,882 | ,22222 | 1,49087 | -2,75123 | 3,19568 |
|  | No Assuming equal variances |  |  | ,149 | 68,373 | ,882 | ,22222 | 1,49087 | -2,75248 | 3,19692 |
| Used_the_Internet_once_week_HighFormalEducation_2015 | Assuming equal variances | 3,598 | ,063 | -116,675 | 60 | ,000 | -90,87097 | ,77884 | -92,42888 | -89,31306 |
|  | No Assuming equal variances |  |  | -116,675 | 54,528 | ,000 | -90,87097 | ,77884 | -92,43210 | -89,30984 |
| Used_the_Internet_once_week_HighFormalEducation_2016 | Assuming equal variances | 6,384 | ,014 | -156,863 | 60 | ,000 | -93,19355 | ,59411 | -94,38194 | -92,00515 |
|  | No Assuming equal variances |  |  | -156,863 | 50,622 | ,000 | -93,19355 | ,59411 | -94,38649 | -92,00061 |
| Used_the_Internet_once_week_HighFormalEducation_2017 | Assuming equal variances | 3,191 | ,078 | -118,532 | 70 | ,000 | -93,13889 | ,78577 | -94,70606 | -91,57172 |
|  | No Assuming equal variances |  |  | -118,532 | 63,184 | ,000 | -93,13889 | ,78577 | -94,70904 | -91,56874 |
| Used_the_Internet_once_week_HighFormalEducation_2018 | Assuming equal variances | ,496 | ,484 | -146,226 | 72 | ,000 | -93,21622 | ,63748 | -94,48701 | -91,94543 |
|  | No Assuming equal variances |  |  | -146,226 | 71,368 | ,000 | -93,21622 | ,63748 | -94,48720 | -91,94523 |
| Used_the_Internet_once_week_HighFormalEducation_2019 | Assuming equal variances | 8,818 | ,004 | -183,325 | 74 | ,000 | -94,73684 | ,51677 | -95,76653 | -93,70716 |
|  | No Assuming equal variances |  |  | -183,325 | 57,765 | ,000 | -94,73684 | ,51677 | -95,77136 | -93,70233 |
| Used_the_Internet_once_week_HighFormalEducation_2020 | Assuming equal variances | 3,736 | ,057 | -221,276 | 70 | ,000 | -95,91667 | ,43347 | -96,78120 | -95,05214 |
|  | No Assuming equal variances |  |  | -221,276 | 66,196 | ,000 | -95,91667 | ,43347 | -96,78207 | -95,05126 |
| Used_the_Internet_once_week_HighFormalEducation_2021 | Assuming equal variances | 3,438 | ,068 | -249,479 | 70 | ,000 | -96,41667 | ,38647 | -97,18746 | -95,64587 |
|  | No Assuming equal variances |  |  | -249,479 | 59,981 | ,000 | -96,41667 | ,38647 | -97,18973 | -95,64360 |

## Tables A-2: Testing H2

### H2 a) Stage 1

| **Test for independent samples** | | | | | | | | | | |
| --- | --- | --- | --- | --- | --- | --- | --- | --- | --- | --- |
|  | | Levene’s test of equality of variances | | t test for mean equality | | | | | | |
|  |  | F | Sig. | t | df | Sig. (two-tailed) | Means difference | Difference standard error | 95% confidence interval of the difference | |
|  |  |  |  |  |  |  |  |  | Lower | Upper |
| 2016_LE_use_desktop | Assuming equal variances | 2,893 | ,095 | 3,130 | 54 | ,003 | 9,643 | 3,080 | 3,467 | 15,819 |
|  | No Assuming equal variances |  |  | 3,130 | 47,281 | ,003 | 9,643 | 3,080 | 3,447 | 15,839 |
| 2018_LE_use_desktop | Assuming equal variances | 2,658 | ,109 | 3,926 | 56 | ,000 | 10,862 | 2,767 | 5,319 | 16,405 |
|  | No Assuming equal variances |  |  | 3,926 | 51,210 | ,000 | 10,862 | 2,767 | 5,308 | 16,416 |
| 2021_LE_use_desktop | Assuming equal variances | 6,382 | ,014 | 4,730 | 56 | ,000 | 12,034 | 2,544 | 6,937 | 17,132 |
|  | No Assuming equal variances |  |  | 4,730 | 48,163 | ,000 | 12,034 | 2,544 | 6,919 | 17,150 |
| 2016_ME_use_desktop | Assuming equal variances | ,236 | ,629 | 2,007 | 54 | ,050 | 6,179 | 3,078 | ,008 | 12,350 |
|  | No Assuming equal variances |  |  | 2,007 | 53,247 | ,050 | 6,179 | 3,078 | ,006 | 12,352 |
| 2018_ME_use_desktop | Assuming equal variances | 1,162 | ,286 | 2,480 | 56 | ,016 | 6,724 | 2,711 | 1,293 | 12,155 |
|  | No Assuming equal variances |  |  | 2,480 | 54,050 | ,016 | 6,724 | 2,711 | 1,289 | 12,159 |
| 2021_ME_use_desktop | Assuming equal variances | ,294 | ,590 | 2,279 | 56 | ,027 | 7,759 | 3,405 | ,937 | 14,580 |
|  | No Assuming equal variances |  |  | 2,279 | 55,525 | ,027 | 7,759 | 3,405 | ,936 | 14,581 |
| 2016_HE_use_desktop | Assuming equal variances | ,213 | ,646 | 1,760 | 54 | ,084 | 5,643 | 3,207 | -,787 | 12,073 |
|  | No Assuming equal variances |  |  | 1,760 | 53,804 | ,084 | 5,643 | 3,207 | -,787 | 12,073 |
| 2018_HE_use_desktop | Assuming equal variances | ,000 | ,991 | 2,886 | 56 | ,006 | 8,448 | 2,928 | 2,583 | 14,313 |
|  | No Assuming equal variances |  |  | 2,886 | 55,925 | ,006 | 8,448 | 2,928 | 2,583 | 14,313 |
| 2021_HE_use_desktop | Assuming equal variances | ,332 | ,567 | 3,777 | 56 | ,000 | 11,000 | 2,912 | 5,166 | 16,834 |
|  | No Assuming equal variances |  |  | 3,777 | 55,637 | ,000 | 11,000 | 2,912 | 5,165 | 16,835 |
| 2016_LE_use_laptop | Assuming equal variances | ,362 | ,550 | ,622 | 54 | ,537 | 3,000 | 4,823 | -6,669 | 12,669 |
|  | No Assuming equal variances |  |  | ,622 | 53,302 | ,537 | 3,000 | 4,823 | -6,672 | 12,672 |
| 2018_LE_use_laptop | Assuming equal variances | ,214 | ,645 | ,950 | 56 | ,346 | 4,448 | 4,681 | -4,929 | 13,826 |
|  | No Assuming equal variances |  |  | ,950 | 55,770 | ,346 | 4,448 | 4,681 | -4,930 | 13,827 |
| 2021_LE_use_laptop | Assuming equal variances | 1,223 | ,273 | ,189 | 56 | ,851 | ,931 | 4,919 | -8,924 | 10,786 |
|  | No Assuming equal variances |  |  | ,189 | 54,672 | ,851 | ,931 | 4,919 | -8,929 | 10,791 |
| 2016_ME_use_laptop | Assuming equal variances | ,068 | ,795 | ,856 | 54 | ,396 | 3,464 | 4,046 | -4,647 | 11,575 |
|  | No Assuming equal variances |  |  | ,856 | 53,991 | ,396 | 3,464 | 4,046 | -4,647 | 11,575 |
| 2018_ME_use_laptop | Assuming equal variances | ,003 | ,954 | ,975 | 56 | ,334 | 3,655 | 3,750 | -3,857 | 11,167 |
|  | No Assuming equal variances |  |  | ,975 | 56,000 | ,334 | 3,655 | 3,750 | -3,857 | 11,167 |
| 2021_ME_use_laptop | Assuming equal variances | ,000 | ,994 | ,518 | 56 | ,607 | 2,000 | 3,862 | -5,737 | 9,737 |
|  | No Assuming equal variances |  |  | ,518 | 55,997 | ,607 | 2,000 | 3,862 | -5,737 | 9,737 |
| 2016_HE_use_laptop | Assuming equal variances | ,173 | ,679 | 1,359 | 54 | ,180 | 3,893 | 2,865 | -1,851 | 9,637 |
|  | No Assuming equal variances |  |  | 1,359 | 53,588 | ,180 | 3,893 | 2,865 | -1,852 | 9,638 |
| 2018_HE_use_laptop | Assuming equal variances | ,261 | ,612 | 1,529 | 56 | ,132 | 4,172 | 2,728 | -1,293 | 9,638 |
|  | No Assuming equal variances |  |  | 1,529 | 55,221 | ,132 | 4,172 | 2,728 | -1,295 | 9,639 |
| 2021_HE_use_laptop | Assuming equal variances | ,043 | ,837 | ,787 | 56 | ,434 | 2,172 | 2,759 | -3,354 | 7,699 |
|  | No Assuming equal variances |  |  | ,787 | 55,993 | ,434 | 2,172 | 2,759 | -3,354 | 7,699 |
| 2016_LE_use_tablet | Assuming equal variances | ,152 | ,698 | ,203 | 54 | ,840 | ,714 | 3,526 | -6,355 | 7,783 |
|  | No Assuming equal variances |  |  | ,203 | 53,637 | ,840 | ,714 | 3,526 | -6,356 | 7,784 |
| 2018_LE_use_tablet | Assuming equal variances | ,189 | ,665 | -,115 | 56 | ,909 | -,379 | 3,293 | -6,975 | 6,217 |
|  | No Assuming equal variances |  |  | -,115 | 55,191 | ,909 | -,379 | 3,293 | -6,977 | 6,219 |
| 2021_LE_use_tablet | Assuming equal variances | ,164 | ,687 | -,358 | 56 | ,722 | -1,034 | 2,889 | -6,821 | 4,752 |
|  | No Assuming equal variances |  |  | -,358 | 55,659 | ,722 | -1,034 | 2,889 | -6,822 | 4,753 |
| 2016_ME_use_tablet | Assuming equal variances | ,002 | ,965 | ,083 | 54 | ,934 | ,357 | 4,291 | -8,246 | 8,960 |
|  | No Assuming equal variances |  |  | ,083 | 53,956 | ,934 | ,357 | 4,291 | -8,246 | 8,960 |
| 2018_ME_use_tablet | Assuming equal variances | ,057 | ,812 | ,190 | 56 | ,850 | ,724 | 3,815 | -6,919 | 8,367 |
|  | No Assuming equal variances |  |  | ,190 | 55,953 | ,850 | ,724 | 3,815 | -6,919 | 8,367 |
| 2021_ME_use_tablet | Assuming equal variances | ,000 | ,991 | ,010 | 56 | ,992 | ,034 | 3,323 | -6,623 | 6,692 |
|  | No Assuming equal variances |  |  | ,010 | 55,986 | ,992 | ,034 | 3,323 | -6,623 | 6,692 |
| 2016_HE_use_tablet | Assuming equal variances | ,086 | ,770 | ,632 | 54 | ,530 | 2,607 | 4,126 | -5,666 | 10,880 |
|  | No Assuming equal variances |  |  | ,632 | 53,948 | ,530 | 2,607 | 4,126 | -5,666 | 10,880 |
| 2018_HE_use_tablet | Assuming equal variances | ,077 | ,783 | ,604 | 56 | ,548 | 2,138 | 3,540 | -4,954 | 9,230 |
|  | No Assuming equal variances |  |  | ,604 | 55,835 | ,548 | 2,138 | 3,540 | -4,954 | 9,230 |
| 2021_HE_use_tablet | Assuming equal variances | ,106 | ,745 | 1,354 | 56 | ,181 | 4,207 | 3,108 | -2,019 | 10,433 |
|  | No Assuming equal variances |  |  | 1,354 | 55,990 | ,181 | 4,207 | 3,108 | -2,019 | 10,433 |
| 2016_LE_use_phone | Assuming equal variances | ,080 | ,778 | 1,157 | 54 | ,252 | 5,643 | 4,876 | -4,133 | 15,419 |
|  | No Assuming equal variances |  |  | 1,157 | 53,640 | ,252 | 5,643 | 4,876 | -4,134 | 15,420 |
| 2018_LE_use_phone | Assuming equal variances | ,203 | ,654 | 1,273 | 56 | ,208 | 5,690 | 4,470 | -3,265 | 14,645 |
|  | No Assuming equal variances |  |  | 1,273 | 55,649 | ,208 | 5,690 | 4,470 | -3,267 | 14,646 |
| 2021_LE_use_phone | Assuming equal variances | ,772 | ,383 | 1,211 | 56 | ,231 | 4,379 | 3,616 | -2,863 | 11,622 |
|  | No Assuming equal variances |  |  | 1,211 | 54,115 | ,231 | 4,379 | 3,616 | -2,869 | 11,628 |
| 2016_ME_use_phone | Assuming equal variances | ,491 | ,486 | ,606 | 54 | ,547 | 2,714 | 4,478 | -6,264 | 11,693 |
|  | No Assuming equal variances |  |  | ,606 | 53,188 | ,547 | 2,714 | 4,478 | -6,268 | 11,696 |
| 2018_ME_use_phone | Assuming equal variances | 1,503 | ,225 | ,164 | 56 | ,870 | ,552 | 3,362 | -6,182 | 7,286 |
|  | No Assuming equal variances |  |  | ,164 | 54,230 | ,870 | ,552 | 3,362 | -6,187 | 7,291 |
| 2021_ME_use_phone | Assuming equal variances | ,538 | ,466 | -,516 | 56 | ,608 | -1,103 | 2,139 | -5,389 | 3,182 |
|  | No Assuming equal variances |  |  | -,516 | 55,404 | ,608 | -1,103 | 2,139 | -5,390 | 3,183 |
| 2016_HE_use_phone | Assuming equal variances | ,947 | ,335 | ,425 | 54 | ,673 | 1,000 | 2,353 | -3,718 | 5,718 |
|  | No Assuming equal variances |  |  | ,425 | 52,580 | ,673 | 1,000 | 2,353 | -3,721 | 5,721 |
| 2018_HE_use_phone | Assuming equal variances | 1,922 | ,171 | ,000 | 56 | 1,000 | ,000 | 1,554 | -3,114 | 3,114 |
|  | No Assuming equal variances |  |  | ,000 | 54,959 | 1,000 | ,000 | 1,554 | -3,115 | 3,115 |
| 2021_HE_use_phone | Assuming equal variances | ,049 | ,826 | -,486 | 56 | ,629 | -,586 | 1,205 | -3,001 | 1,829 |
|  | No Assuming equal variances |  |  | -,486 | 55,999 | ,629 | -,586 | 1,205 | -3,001 | 1,829 |

### H2 b) Stage 2

| **Test for independent samples** | | | | | | | | | | |
| --- | --- | --- | --- | --- | --- | --- | --- | --- | --- | --- |
|  | | Levene’s test of equality of variances | | t test for mean equality | | | | | | |
|  |  | F | Sig. | t | df | Sig. (two-tailed) | Means difference | Difference standard error | 95% confidence interval of the difference | |
|  |  |  |  |  |  |  |  |  | Lower | Upper |
| 2016_LE_use_interntet_3_months | Assuming equal variances | ,309 | ,581 | 1,335 | 54 | ,188 | 6,857 | 5,138 | -3,443 | 17,158 |
|  | No Assuming equal variances |  |  | 1,335 | 53,180 | ,188 | 6,857 | 5,138 | -3,447 | 17,161 |
| 2018_LE_use_interntet_3_months | Assuming equal variances | ,566 | ,455 | 1,424 | 56 | ,160 | 6,655 | 4,674 | -2,708 | 16,018 |
|  | No Assuming equal variances |  |  | 1,424 | 55,188 | ,160 | 6,655 | 4,674 | -2,711 | 16,021 |
| 2021_LE_use_interntet_3_months | Assuming equal variances | 2,063 | ,157 | 1,192 | 56 | ,238 | 4,621 | 3,878 | -3,147 | 12,388 |
|  | No Assuming equal variances |  |  | 1,192 | 53,253 | ,239 | 4,621 | 3,878 | -3,156 | 12,397 |
| 2016_ME_use_interntet_3_months | Assuming equal variances | ,281 | ,598 | ,451 | 54 | ,654 | 1,321 | 2,932 | -4,556 | 7,199 |
|  | No Assuming equal variances |  |  | ,451 | 53,413 | ,654 | 1,321 | 2,932 | -4,557 | 7,200 |
| 2018_ME_use_interntet_3_months | Assuming equal variances | ,278 | ,600 | ,127 | 56 | ,899 | ,310 | 2,441 | -4,579 | 5,199 |
|  | No Assuming equal variances |  |  | ,127 | 55,726 | ,899 | ,310 | 2,441 | -4,579 | 5,200 |
| 2021_ME_use_interntet_3_months | Assuming equal variances | ,289 | ,593 | -,043 | 56 | ,966 | -,069 | 1,601 | -3,276 | 3,138 |
|  | No Assuming equal variances |  |  | -,043 | 55,173 | ,966 | -,069 | 1,601 | -3,277 | 3,139 |
| 2016_HE_use_interntet_3_months | Assuming equal variances | ,021 | ,885 | -,149 | 54 | ,882 | -,107 | ,717 | -1,545 | 1,331 |
|  | No Assuming equal variances |  |  | -,149 | 53,973 | ,882 | -,107 | ,717 | -1,545 | 1,331 |
| 2018_HE_use_interntet_3_months | Assuming equal variances | ,017 | ,898 | ,201 | 56 | ,841 | ,138 | ,686 | -1,236 | 1,512 |
|  | No Assuming equal variances |  |  | ,201 | 55,949 | ,841 | ,138 | ,686 | -1,236 | 1,512 |
| 2021_HE_use_interntet_3_months | Assuming equal variances | ,044 | ,835 | -,072 | 56 | ,943 | -,034 | ,481 | -,998 | ,929 |
|  | No Assuming equal variances |  |  | -,072 | 55,576 | ,943 | -,034 | ,481 | -,999 | ,930 |
| 2016_LE_use_interntet_daily_inlastmonths | Assuming equal variances | 1,679 | ,201 | ,878 | 54 | ,384 | 1,821 | 2,074 | -2,336 | 5,979 |
|  | No Assuming equal variances |  |  | ,878 | 53,068 | ,384 | 1,821 | 2,074 | -2,338 | 5,981 |
| 2018_LE_use_interntet_daily_inlastmonths | Assuming equal variances | 1,301 | ,259 | ,699 | 56 | ,487 | 1,414 | 2,022 | -2,638 | 5,465 |
|  | No Assuming equal variances |  |  | ,699 | 55,062 | ,487 | 1,414 | 2,022 | -2,639 | 5,467 |
| 2021_LE_use_interntet_daily_inlastmonths | Assuming equal variances | ,312 | ,579 | ,379 | 56 | ,706 | ,621 | 1,637 | -2,658 | 3,899 |
|  | No Assuming equal variances |  |  | ,379 | 55,821 | ,706 | ,621 | 1,637 | -2,658 | 3,900 |
| 2016_ME_use_interntet_daily_inlastmonths | Assuming equal variances | ,198 | ,658 | ,783 | 54 | ,437 | 1,643 | 2,098 | -2,564 | 5,849 |
|  | No Assuming equal variances |  |  | ,783 | 53,880 | ,437 | 1,643 | 2,098 | -2,564 | 5,850 |
| 2018_ME_use_interntet_daily_inlastmonths | Assuming equal variances | ,000 | ,992 | ,514 | 56 | ,609 | ,897 | 1,743 | -2,595 | 4,389 |
|  | No Assuming equal variances |  |  | ,514 | 55,944 | ,609 | ,897 | 1,743 | -2,596 | 4,389 |
| 2021_ME_use_interntet_daily_inlastmonths | Assuming equal variances | ,031 | ,861 | ,000 | 56 | 1,000 | ,000 | 1,428 | -2,860 | 2,860 |
|  | No Assuming equal variances |  |  | ,000 | 55,856 | 1,000 | ,000 | 1,428 | -2,860 | 2,860 |
| 2016_HE_use_interntet_daily_inlastmonths | Assuming equal variances | 1,821 | ,183 | 1,212 | 54 | ,231 | ,964 | ,796 | -,631 | 2,560 |
|  | No Assuming equal variances |  |  | 1,212 | 52,133 | ,231 | ,964 | ,796 | -,633 | 2,561 |
| 2018_HE_use_interntet_daily_inlastmonths | Assuming equal variances | ,233 | ,631 | 1,721 | 56 | ,091 | 1,069 | ,621 | -,176 | 2,314 |
|  | No Assuming equal variances |  |  | 1,721 | 55,519 | ,091 | 1,069 | ,621 | -,176 | 2,314 |
| 2021_HE_use_interntet_daily_inlastmonths | Assuming equal variances | ,427 | ,516 | -,070 | 56 | ,945 | -,034 | ,496 | -1,028 | ,959 |
|  | No Assuming equal variances |  |  | -,070 | 55,672 | ,945 | -,034 | ,496 | -1,028 | ,959 |

### H2 c) Stage 3

| **Test for independent samples** | | | | | | | | | | |
| --- | --- | --- | --- | --- | --- | --- | --- | --- | --- | --- |
|  | | Levene’s test of equality of variances | | t test for mean equality | | | | | | |
|  |  | F | Sig. | t | df | Sig. (two-tailed) | Means difference | Difference standard error | 95% confidence interval of the difference | |
|  |  |  |  |  |  |  |  |  | Lower | Upper |
| 2016_LE_use_E-gover_send_forms | Assuming equal variances | ,089 | ,767 | ,538 | 54 | ,593 | 2,500 | 4,644 | -6,810 | 11,810 |
|  | No Assuming equal variances |  |  | ,538 | 53,999 | ,593 | 2,500 | 4,644 | -6,810 | 11,810 |
| 2018_LE_use_E-gover_send_forms | Assuming equal variances | ,001 | ,970 | ,416 | 56 | ,679 | 2,276 | 5,470 | -8,682 | 13,233 |
|  | No Assuming equal variances |  |  | ,416 | 55,963 | ,679 | 2,276 | 5,470 | -8,682 | 13,234 |
| 2021_LE_use_E-gover_send_forms | Assuming equal variances | ,134 | ,716 | ,348 | 56 | ,729 | 2,034 | 5,838 | -9,661 | 13,730 |
|  | No Assuming equal variances |  |  | ,348 | 55,927 | ,729 | 2,034 | 5,838 | -9,661 | 13,730 |
| 2016_ME_use_E-gover_send_forms | Assuming equal variances | ,529 | ,470 | ,580 | 54 | ,564 | 3,143 | 5,417 | -7,717 | 14,003 |
|  | No Assuming equal variances |  |  | ,580 | 53,683 | ,564 | 3,143 | 5,417 | -7,718 | 14,004 |
| 2018_ME_use_E-gover_send_forms | Assuming equal variances | ,046 | ,831 | ,447 | 56 | ,656 | 2,690 | 6,015 | -9,360 | 14,739 |
|  | No Assuming equal variances |  |  | ,447 | 55,919 | ,656 | 2,690 | 6,015 | -9,360 | 14,739 |
| 2021_ME_use_E-gover_send_forms | Assuming equal variances | ,102 | ,750 | ,349 | 56 | ,728 | 2,034 | 5,825 | -9,634 | 13,703 |
|  | No Assuming equal variances |  |  | ,349 | 55,954 | ,728 | 2,034 | 5,825 | -9,634 | 13,703 |
| 2016_HE_use_E-gover_send_forms | Assuming equal variances | ,234 | ,631 | ,793 | 54 | ,431 | 4,286 | 5,402 | -6,545 | 15,116 |
|  | No Assuming equal variances |  |  | ,793 | 53,937 | ,431 | 4,286 | 5,402 | -6,545 | 15,117 |
| 2018_HE_use_E-gover_send_forms | Assuming equal variances | ,152 | ,698 | ,717 | 56 | ,476 | 4,034 | 5,626 | -7,236 | 15,305 |
|  | No Assuming equal variances |  |  | ,717 | 55,978 | ,476 | 4,034 | 5,626 | -7,236 | 15,305 |
| 2021_HE_use_E-gover_send_forms | Assuming equal variances | ,019 | ,891 | ,740 | 56 | ,462 | 3,379 | 4,566 | -5,768 | 12,527 |
|  | No Assuming equal variances |  |  | ,740 | 55,976 | ,462 | 3,379 | 4,566 | -5,768 | 12,527 |
| 2016_LE_High_dig_skills | Assuming equal variances | ,001 | ,974 | 1,023 | 54 | ,311 | 3,750 | 3,667 | -3,603 | 11,103 |
|  | No Assuming equal variances |  |  | 1,023 | 53,824 | ,311 | 3,750 | 3,667 | -3,603 | 11,103 |
| 2019_LE_High_dig_skills | Assuming equal variances | ,461 | ,500 | 1,438 | 56 | ,156 | 4,931 | 3,429 | -1,939 | 11,801 |
|  | No Assuming equal variances |  |  | 1,438 | 55,724 | ,156 | 4,931 | 3,429 | -1,940 | 11,802 |
| 2021_LE_High_dig_skills | Assuming equal variances | ,283 | ,597 | ,814 | 56 | ,419 | 2,207 | 2,711 | -3,224 | 7,638 |
|  | No Assuming equal variances |  |  | ,814 | 55,606 | ,419 | 2,207 | 2,711 | -3,225 | 7,638 |
| 2016_ME_use_High_dig_skills | Assuming equal variances | ,666 | ,418 | 1,054 | 54 | ,297 | 3,429 | 3,254 | -3,096 | 9,953 |
|  | No Assuming equal variances |  |  | 1,054 | 53,359 | ,297 | 3,429 | 3,254 | -3,098 | 9,955 |
| 2019_ME_use_High_dig_skills | Assuming equal variances | ,950 | ,334 | 1,042 | 56 | ,302 | 3,517 | 3,377 | -3,248 | 10,282 |
|  | No Assuming equal variances |  |  | 1,042 | 54,412 | ,302 | 3,517 | 3,377 | -3,252 | 10,287 |
| 2021_ME_use_High_dig_skills | Assuming equal variances | 1,082 | ,303 | 1,390 | 56 | ,170 | 4,448 | 3,201 | -1,963 | 10,860 |
|  | No Assuming equal variances |  |  | 1,390 | 54,784 | ,170 | 4,448 | 3,201 | -1,966 | 10,863 |
| 2016_HE_use_High_dig_skills | Assuming equal variances | ,001 | ,972 | -2,043 | 54 | ,046 | -3,107 | 1,521 | -6,156 | -,058 |
|  | No Assuming equal variances |  |  | -2,043 | 53,757 | ,046 | -3,107 | 1,521 | -6,156 | -,058 |
| 2019_HE_use_High_dig_skills | Assuming equal variances | ,834 | ,365 | -2,406 | 56 | ,019 | -3,793 | 1,576 | -6,951 | -,635 |
|  | No Assuming equal variances |  |  | -2,406 | 55,950 | ,019 | -3,793 | 1,576 | -6,951 | -,635 |
| 2021_HE_use_High_dig_skills | Assuming equal variances | ,318 | ,575 | 2,662 | 56 | ,010 | 7,862 | 2,954 | 1,945 | 13,779 |
|  | No Assuming equal variances |  |  | 2,662 | 55,542 | ,010 | 7,862 | 2,954 | 1,944 | 13,780 |
| 2016_Employed_with ICT education | Assuming equal variances | ,019 | ,891 | 35,161 | 55 | ,000 | 62,76724 | 1,78512 | 59,18978 | 66,34470 |
|  | No Assuming equal variances |  |  | 35,151 | 54,849 | ,000 | 62,76724 | 1,78565 | 59,18849 | 66,34599 |
| 2019_Employed_with ICT education | Assuming equal variances | ,002 | ,965 | 46,703 | 53 | ,000 | 65,98103 | 1,41279 | 63,14733 | 68,81474 |
|  | No Assuming equal variances |  |  | 46,715 | 52,407 | ,000 | 65,98103 | 1,41241 | 63,14735 | 68,81471 |
| 2021_Employed_with ICT education | Assuming equal variances | ,000 | 1,000 | 32,385 | 52 | ,000 | 62,93333 | 1,94331 | 59,03379 | 66,83288 |
|  | No Assuming equal variances |  |  | 32,385 | 52,000 | ,000 | 62,93333 | 1,94331 | 59,03379 | 66,83288 |
